# Medicinal Plant Polysaccharides as Microbiota-Directed Modulators of Immunosenescence: Structural Determinants, Metabolite Reprogramming, and Host Immune Regulation

**DOI:** 10.3390/nu18162641

**Published:** 2026-08-12

**Authors:** Kailang Mu, Meihui He, Ruiqi Liao, Changliu Shao, Pingxuan Xie, Junli Xie, Yuchen Liu, Zhigang Ju, Ke Zhong, Yuan Yuan, Yuxin Pang

**Affiliations:** 1 College of Pharmacy, Guizhou University of Traditional Chinese Medicine, Guiyang 550025, China; mkl980818@163.com (K.M.); hemeihui@stu.gzy.edu.cn (M.H.); m18285888952_1@163.com (R.L.); shaochangliu178@163.com (C.S.); xiepingxuan@stu.gzy.edu.cn (P.X.); xiejunli1008@163.com (J.X.); lyc8564732@163.com (Y.L.); juzhigangz@163.com (Z.J.); lionzhongke@163.com (K.Z.); 2 College of Pharmacy, Guangdong Medical University, Dongguan 523808, China; 3 College of Life Sciences and Biopharmaceuticals, Guangdong Medical University, Guangzhou 510006, China

**Keywords:** medicinal plant polysaccharides, gut microbiota, immunosenescence, inflammaging, nutritional immunology, microbial metabolites, polysaccharide utilization loci, healthy aging

## Abstract

Immunosenescence is a major contributor to age-associated morbidity, yet microbiota-directed strategies capable of restoring immune homeostasis remain insufficiently validated. Medicinal plant polysaccharides (MPPs) are structurally diverse macromolecules that often resist host digestion and undergo microbial transformation in the colon, but their effects cannot be interpreted as those of a homogeneous intervention class. In this structured narrative review, we critically synthesize evidence across structural carbohydrate biology, microbial ecology, metabolite signaling, and immune aging and propose a structure–microbiota–metabolite–immunity framework for evaluating how MPPs may influence immunosenescence. Monosaccharide composition, glycosidic linkages, molecular-weight distribution, branching, uronic acid content, chemical substitutions, and higher-order conformation can shape microbial carbohydrate-active enzyme activity, polysaccharide utilization, and ecological cross-feeding. The resulting changes in short-chain fatty acids, secondary bile acids, tryptophan-derived indoles, and other metabolites may affect epithelial barrier integrity, regulatory T-cell/T helper 17-cell (Treg/Th17) balance, macrophage polarization, nuclear factor-κB (NF-κB) signaling, NLR family pyrin domain-containing 3 (NLRP3) inflammasome activation, and systemic inflammatory tone. However, evidence from in vitro systems and young disease models primarily supports mechanistic plausibility and should not be treated as direct evidence of immunosenescence modification. Translation will require structurally defined preparations, causal validation in aging-relevant models, comparison with established fermentable fibers, identification of responder phenotypes, and adequately powered trials in older adults.

## 1. Introduction

Global population aging has become one of the defining demographic transitions of the twenty-first century. By 2050, the number of individuals aged 60 years and older is projected to reach 2.1 billion worldwide, accounting for approximately 22% of the global population [1]. Aging is accompanied by a progressive decline in immune competence, a process broadly referred to as immunosenescence, which is characterized by impaired adaptive immune responsiveness, dysregulated innate immunity, and a persistent state of chronic low-grade inflammation known as inflammaging [2]. These processes contribute to impaired vaccine responsiveness [3], increased susceptibility to infection [4], and the development of age-related disorders, including cardiovascular disease, cancer, and neurodegenerative diseases [5].

Immunosenescence affects both adaptive and innate immunity. Thymic involution reduces naïve T-cell output, while repeated antigenic stimulation promotes the accumulation of memory and terminally differentiated T cells, contraction of the T-cell receptor repertoire, and acquisition of senescence-associated phenotypes [6]. Aging also impairs the phagocytic and antimicrobial functions of neutrophils and macrophages while increasing basal production of interleukin-6, tumor necrosis factor-α, and interleukin-1β [7]. These alterations weaken immune responsiveness and sustain tissue-damaging inflammation, linking immune aging to dysfunction across multiple organ systems [8].

The gut microbiota is increasingly recognized as an important interface between aging, barrier dysfunction, and systemic immunity. The human intestine contains approximately 10^13^–10^14^ microbial cells whose collective metabolic activities influence epithelial integrity, mucosal immunity, and inflammatory tone [9]. Aging does not produce a single, universal microbial signature; instead, microbiota remodeling varies with diet, medication exposure, geography, frailty, and comorbidity. Nevertheless, several recurrent patterns have been reported. At the phylum level, some older and frail populations show a relative shift from obligate anaerobic Firmicutes toward facultative Proteobacteria. At the genus level, reductions in *Faecalibacterium*, *Bifidobacterium*, and *Akkermansia* may be accompanied by expansion of opportunistic or pro-inflammatory taxa [10,11,12]. At the species and functional levels, depletion of butyrate-producing organisms, including *Faecalibacterium prausnitzii* and selected *Roseburia* species, may reduce short-chain fatty acid production, impair tight-junction maintenance, and promote a self-reinforcing gut permeability–endotoxemia–inflammaging axis. Aging-related microbial remodeling may also alter secondary bile acid metabolism and redirect tryptophan metabolism away from potentially immunoregulatory indoles. Experimental microbiota-transfer studies support a contributory role for the gut microbiota in immune aging: microbiota from aged donors can transmit selected inflammatory or functional phenotypes to younger recipients, whereas transfer from younger donors can ameliorate some age-associated abnormalities in animal models [13,14,15]. These effects are model-dependent, however, and should not be interpreted as evidence of clinical efficacy in humans.

MPPs comprise chemically diverse macromolecules isolated from medicinal plants and medicinal fungi, including *Astragalus mongholicus*, *Astragalus membranaceus*, *Ganoderma lucidum*, *Lycium barbarum*, and *Panax ginseng*. The term ‘MPPs’ is used here as an operational umbrella and does not imply a homogeneous intervention class. β-Glucans, arabinogalactans, pectic polysaccharides, glucomannans, galactomannans, and polysaccharide–protein complexes differ in backbone composition, glycosidic linkages, branching, charge density, molecular size, conformation, solubility, microbial degradability, and receptor-interactive capacity. Many resist complete digestion by host enzymes and therefore become available for microbial transformation in the colon, although fermentability varies substantially among classes and preparations [16,17]. Their activity has traditionally been attributed to direct interactions with host pattern-recognition receptors [18], but microbial degradation, ecological cross-feeding, and metabolite production are increasingly recognized as additional contributors [19,20,21].

**Figure 1:**
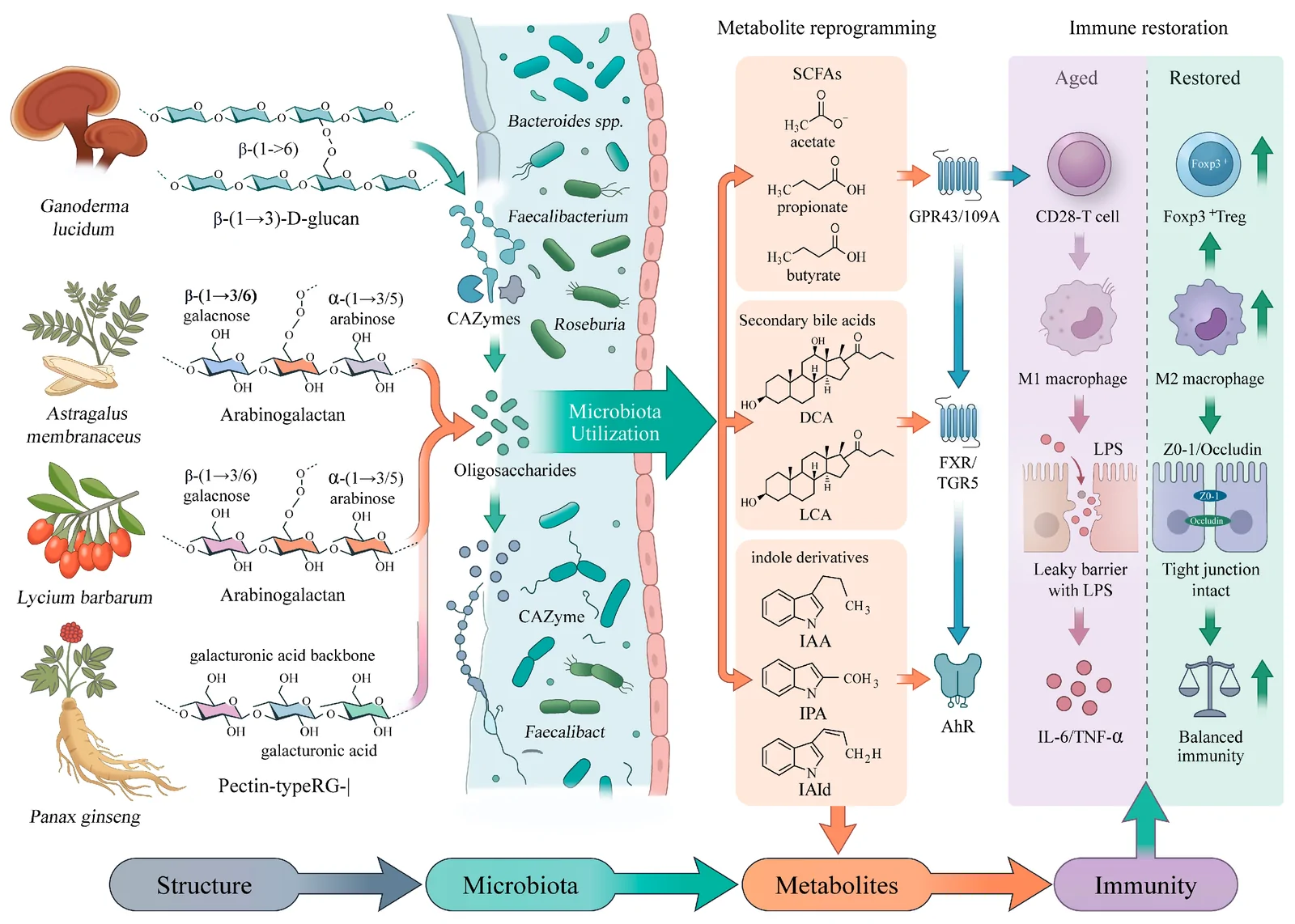
Schematic illustration of the structure–microbiota–metabolite–immunity axis through which MPPs may function as microbiota-directed modulators of immunosenescence. Abbreviations: AhR, aryl hydrocarbon receptor; CAZymes, carbohydrate-active enzymes; GPR, G-protein-coupled receptor; LPS, lipopolysaccharide; MPPs, medicinal plant polysaccharides; SCFAs, short-chain fatty acids; Treg, regulatory T cell. This schematic was created by the authors using Adobe Illustrator 2026 (Adobe Inc., San Jose, CA, USA); no panel, icon arrangement, or graphical composition was reproduced or adapted from a published source.

This review therefore evaluates MPPs as structurally defined, microbiota-directed substrates rather than as interchangeable immune stimulants. We examine how monosaccharide composition, glycosidic linkages, molecular-weight distribution, branching, uronic acid content, chemical substitutions, and higher-order conformation influence microbial carbohydrate-active enzymes (CAZymes) and polysaccharide utilization loci (PULs). We then assess how changes in short-chain fatty acids (SCFAs), secondary bile acids (SBAs), and tryptophan-derived indoles may affect epithelial barrier function, regulatory T-cell/T helper 17-cell (Treg/Th17) balance, macrophage polarization, inflammasome signaling, and systemic inflammation (Figure 1). Throughout, mechanistic evidence is distinguished from findings obtained in aging-relevant models, and MPPs are critically compared with conventional fermentable fibers. This framework is used to identify the priorities for structural standardization, causal validation, responder stratification, and clinical translation.

## 2. Methods

### 2.1. Literature Search and Eligibility

A structured narrative review was conducted to identify and synthesize literature relevant to the structural characteristics of MPPs, microbiota-mediated metabolism, and their potential roles in immune aging. Literature searches were performed in PubMed/MEDLINE, Scopus, and the Web of Science Core Collection for publications from January 2000 to 10 June 2026. The search strategy combined terms related to MPPs, medicinal fungi-derived polysaccharides, gut microbiota or microbiome, CAZymes, PULs, microbial fermentation, SCFAs, SBAs, tryptophan-derived metabolites, immunosenescence, inflammaging, intestinal barrier function, macrophage polarization, and inflammatory signaling pathways. The initial search identified approximately 6500 records across the three databases. After removal of duplicates and preliminary screening based on titles and abstracts, approximately 1200 publications were considered potentially relevant. Full-text evaluation was subsequently performed according to predefined conceptual and mechanistic criteria, and approximately 267 publications were incorporated into the final narrative synthesis. Additional studies were identified through manual screening of reference lists from key reviews and primary research articles. Eligible studies included peer-reviewed original articles, systematic reviews, meta-analyses, and mechanistic investigations addressing MPP structural characterization, microbial degradation or fermentation, microbiota-derived metabolites, immune regulation, intestinal barrier function, or aging-related processes. Priority was given to studies involving older adults, naturally aged animals, or accelerated-aging models. Evidence derived from in vitro fermentation systems, young disease models, or isolated cellular experiments was interpreted primarily as mechanistic support rather than direct evidence of immunosenescence modification. Further details of the literature-search strategy, inclusion and exclusion criteria, study selection, data extraction, and evidence-appraisal approach are provided in Supplementary Method S1.

### 2.2. Evidence Synthesis and Appraisal

Eligible sources included peer-reviewed original studies, systematic reviews, meta-analyses, and mechanistic investigations addressing MPP structural characterization, microbial degradation or fermentation, microbiota-derived metabolites, intestinal barrier function, immune cell phenotypes, inflammatory mediators, or aging-related outcomes. For each study, we extracted the polysaccharide source and fraction, available structural descriptors, experimental model, intervention characteristics, microbiota and metabolite findings, immune endpoints, and causal testing strategies. Human studies in older adults and studies using naturally aged or accelerated-aging animals were given the greatest interpretive weight. Evidence from young disease models, in vitro fermentation systems, defined microbial cultures, or isolated immune cell experiments was used to support mechanistic interpretation rather than as direct evidence of immunosenescence modification. Evidence was qualitatively appraised according to structural definition, microbiota and metabolite resolution, causal testing, aging relevance, reproducibility, and translational value. The operational definitions and interpretive criteria used for the strong, moderate, and weak evidence tiers are provided in Table S3.

## 3. Structural Diversity of MPPs and Their Specificity as Microbial Substrates

### 3.1. Major Structural Types and Sources

The structural diversity of MPPs provides the molecular basis for their substrate specificity toward the gut microbiota and their downstream immunoregulatory activities. According to monosaccharide composition and glycosidic linkage patterns, MPPs can be broadly classified into homopolysaccharides and heteropolysaccharides, each of which comprises multiple structural subtypes. These structural attributes strongly influence their accessibility to microbial carbohydrate-degrading systems and thereby shape their selective utilization by gut microorganisms [17].

β-Glucans are among the most extensively studied homopolysaccharides and are predominantly found in medicinal fungi. *Ganoderma lucidum* polysaccharides (GLPs) are typically characterized by a β-(1→3)-D-glucan backbone substituted with β-(1→6)-D-glucan side chains, with molecular weights ranging from approximately 10 to 1000 kDa. Their triple-helical conformation is considered an important structural determinant of bioactivity [22]. Lentinan is another representative β-(1→3)-D-glucan, containing approximately two β-(1→6) branches per five glucose residues and exhibiting a molecular weight of about 500 kDa [23]. Poria polysaccharides also possess a β-(1→3)-D-glucan backbone, although their branching degree and molecular weight distribution differ from those of GLP and lentinan [24]. In contrast, β-glucans from oats and barley predominantly contain mixed β-(1→3,1→4)-D-glucan linkages and are generally more readily degraded by specific microbial taxa [25].

Arabinogalactans (AGs) represent an important class of plant-derived heteropolysaccharides. The major active fraction of Astragalus polysaccharides (APSs) is enriched in AGs, which commonly contain β-(1→3)- and β-(1→6)-linked galactan backbones decorated with α-(1→3)- and α-(1→5)-linked arabinose side chains, with molecular weights typically ranging from 3 to 40 kDa [26]. *Lycium barbarum* polysaccharides (LBPs) are also rich in AG-type structures, with an arabinose-to-galactose ratio of approximately 1:1.5 and minor amounts of rhamnose and glucuronic acid [27]. Angelica polysaccharides possess more complex AG side chains containing multiple glycosidic linkage types [28].

Pectic polysaccharides are widely distributed in medicinal plant cell walls and are characterized by galacturonic acid-rich domains. Ginseng polysaccharides (GPSs) contain homogalacturonan regions composed of α-(1→4)-linked galacturonic acid, together with rhamnogalacturonan type I and type II domains. Their rhamnogalacturonan-I (RG-I) side chains are enriched in arabinose and galactose [29]. Yam polysaccharides also contain abundant pectic components, and their degree of methyl esterification can affect their biological activity [30].

Glucomannans and galactomannans constitute another important group of heteropolysaccharides. Konjac glucomannan is composed of β-(1→4)-linked glucose and mannose at an approximate ratio of 1:1.6, with molecular weights that may reach 200–2000 kDa [31]. Guar gum galactomannan consists of a β-(1→4)-linked mannan backbone substituted with α-(1→6)-linked galactose side chains, with a galactose-to-mannose ratio of approximately 1:2 [32].

Polysaccharide–protein complexes represent a special structural form of MPPs, in which the protein content usually ranges from 5% to 30%. Representative examples include glycopeptide or protein-bound polysaccharide fractions from medicinal fungi, such as GLP-associated peptides and polysaccharopeptide (PSP)-type complexes. In these macromolecular assemblies, the protein moiety may enhance immunological activity through glycopeptide bonds or non-covalent interactions with the polysaccharide backbone [33]. Table 1 summarizes MPPs at the structural-class level, emphasizing that β-glucans, arabinogalactans, pectic/RG-I polysaccharides, glucomannans/galactomannans, conjugated complexes, and chemically modified MPPs should not be interpreted as interchangeable substrates or equivalent immunological interventions.

### 3.2. Association Between Structural Features and Microbial Utilization

The structural features of MPPs strongly influence their utilization by the gut microbiota, and this structure-dependent microbial utilization is central to understanding their immunoregulatory mechanisms [34]. Microbial degradation of complex polysaccharides depends on genome-encoded CAZymes, particularly glycoside hydrolases (GHs), polysaccharide lyases, carbohydrate esterases, and associated substrate-binding proteins. Differences in CAZyme repertoires among microbial genera confer highly specific glycan-degradation capacities [35].

Glycosidic linkage type is one of the primary determinants of microbial utilization. Degradation of β-(1→3) linkages requires GH16, GH55, and GH81 family enzymes, which are frequently encoded by *Bacteroides* species. Accordingly, Ganoderma β-glucans have been reported to enrich *Bacteroides thetaiotaomicron* and *Bacteroides uniformis* [36]. By contrast, β-(1→4) linkages are mainly degraded by GH5 and GH9 family enzymes, which are more abundant in genera such as *Ruminococcus* and *Roseburia*. Consistent with this enzymatic specificity, konjac glucomannan is preferentially utilized by butyrate-producing bacteria such as *Faecalibacterium prausnitzii* and *Roseburia intestinalis* [37].

Branching architecture also exerts a major influence on microbial accessibility. Highly branched polysaccharides require coordinated debranching before backbone degradation can proceed efficiently. In AGs, arabinose-containing side chains are removed by GH43- and GH51-family α-L-arabinofuranosidases. The polysaccharide utilization loci (PULs) of Bacteroides ovatus provide an integrated enzymatic system capable of degrading these complex side-chain structures [38].

Molecular weight further regulates substrate accessibility and degradation efficiency. High-molecular-weight polysaccharides, particularly those exceeding 100 kDa, generally require initial extracellular cleavage by polysaccharide lyases or endo-glycoside hydrolases. *Bacteroides* species can adopt a “selfish” utilization strategy, in which SusD-like proteins capture complex glycans at the cell surface and facilitate their efficient degradation and import [39]. In contrast, low-molecular-weight oligosaccharides below approximately 10 kDa may be more directly utilized by genera such as Bifidobacterium and Lactobacillus [40].

Uronic acid content and chemical substituents are particularly important for pectic and modified polysaccharides. Uronic acid-rich domains select for bacteria equipped with pectate lyases, glycoside hydrolases, and carbohydrate esterases; for example, *Bacteroides dorei* and *Faecalibacterium prausnitzii* have been identified as important degraders of ginseng pectic polysaccharides [41]. Structural modifications can further change microbial access. Sulfation increases negative charge and hydration, which can improve solubility and receptor interaction but may also require sulfatase-positive microbial guilds before backbone hydrolysis proceeds. Acetylation and methyl esterification can sterically shield glycosidic bonds or uronic acid residues, slowing degradation unless carbohydrate esterases remove these groups. Conversely, controlled deacetylation, desulfation, or partial hydrolysis can expose linkages, alter molecular size, and shift fermentation toward different SCFA ratios. Thus, sulfation, acetylation, methyl esterification, and processing-induced fragmentation should be reported not only as chemical descriptors but also as potential determinants of CAZyme accessibility, PUL activation, fermentation kinetics, and downstream immune signaling [42,43,44].

### 3.3. Comparison with Conventional Dietary Fibers and Synthetic Prebiotics

Compared with conventional synthetic or semi-synthetic prebiotics, such as inulin and fructooligosaccharides, MPPs may provide broader functional potential because of their greater structural complexity, more diverse microbial utilization patterns, and wider range of metabolite outputs. Synthetic prebiotics generally possess relatively simple and repetitive structures and primarily enrich Bifidobacterium and selected Lactobacillus species, resulting in a comparatively restricted microbial response [45]. In contrast, many MPPs are complex heteropolysaccharides composed of multiple monosaccharides, diverse glycosidic linkages, branched domains, uronic acids, and occasionally protein or phenolic conjugates. These features allow them to engage a wider range of microbial degraders and cross-feeding partners, thereby generating broader ecological effects within the gut microbial community [46].

Differences in primary degraders further distinguish MPPs from conventional prebiotics. Inulin utilization largely depends on GH32-family β-fructofuranosidases, particularly those encoded by Bifidobacterium. However, the abundance and functional capacity of Bifidobacterium often decline in older adults, which may limit the efficacy of inulin-based interventions in aged populations [11]. By contrast, MPPs may engage relatively retained glycan-degradation capacities in the aged gut microbiota, including those associated with Bacteroides and other primary degraders, while indirectly supporting beneficial butyrate-producing taxa such as Faecalibacterium through cross-feeding networks [47].

Cross-feeding capacity represents another important advantage of MPPs. Their degradation typically generates multiple intermediate oligosaccharides and monosaccharides, which can support a cascade of microbial interactions and lead to more balanced production of metabolites, including butyrate [48]. Synthetic prebiotics, in contrast, are often converted mainly into acetate and lactate, and their secondary utilization pathways may be comparatively limited [49]. Moreover, MPP fermentation can generate a more diverse metabolite spectrum, including SCFAs, tryptophan-derived indoles, phenolic acid metabolites, and bile acid-modulating outputs, thereby providing broader opportunities for immunomodulation [50].

**Figure 2:**
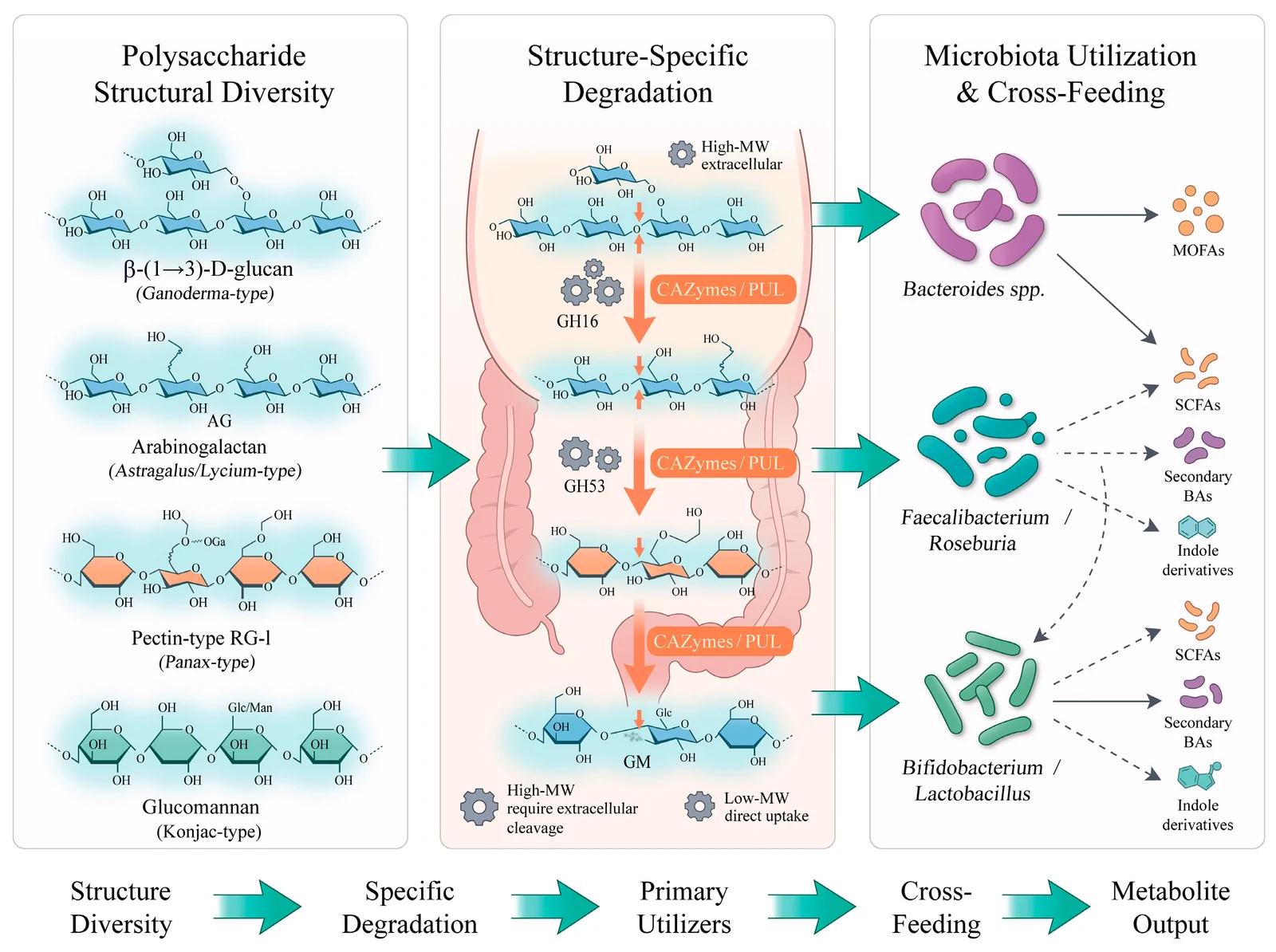
Structure-dependent microbial utilization of MPPs and downstream metabolite output. The structural diversity of MPPs, including monosaccharide composition, glycosidic linkage patterns, molecular weight distribution, branching architecture, uronic acid content, and chemical modifications, determines their recognition and degradation by gut microbial CAZymes and PULs. Primary degraders generate intermediate oligosaccharides and monosaccharides that support microbial cross-feeding networks, ultimately leading to the production of immunoregulatory metabolites, including SCFAs, bile acid derivatives, and tryptophan-derived indoles. Abbreviations: CAZymes, carbohydrate-active enzymes; MPPs, medicinal plant polysaccharides; PULs, polysaccharide utilization loci; SCFAs, short-chain fatty acids. This schematic was created by the authors using Adobe Illustrator 2026 (Adobe Inc., San Jose, CA, USA); no panel, icon arrangement, or graphical composition was reproduced or adapted from a published source.

The potential adaptability of MPPs to the aged gut microbiota supports further evaluation as microbiota-directed interventions for immunosenescence. In an elderly constipation cohort, APS supplementation was associated with increased fecal butyrate and *Faecalibacterium* abundance [51]. Because this was a disease-specific study and did not use immune-aging endpoints or a head-to-head comparison with a standardized fermentable fiber, it does not establish efficacy against immunosenescence. In addition to microbiota-mediated activity, MPPs may exert complementary effects through direct activation of pattern-recognition receptors such as Dectin-1, bioactivity of fermentation-derived oligosaccharides, metabolite reprogramming, and remodeling of microbial ecological networks [21] (Figure 2). Although structural heterogeneity and quality-control challenges remain major obstacles to clinical translation, advances in chromatographic separation, mass spectrometry, nuclear magnetic resonance spectroscopy, and multi-omics analysis are progressively improving the standardization and mechanistic characterization of MPPs.

Conventional fibers currently have important translational advantages over most MPPs: they are better standardized, more widely consumed, supported by larger human trials, and embedded in dietary guidelines. By contrast, MPP studies often use heterogeneous extracts with incomplete structural annotation, variable purity, limited batch-to-batch comparability, and short intervention periods. Consequently, the most balanced translational position is that MPPs are complementary microbiota-directed candidates, especially for precision formulations, rather than replacements for established fermentable fibers.

The key question is not whether MPPs produce SCFAs—many fermentable fibers do—but whether defined MPP structures reproducibly produce additional or more targeted microbiota, metabolite, barrier, and immune-aging effects beyond those achieved by conventional fibers in older adults.

**Table 1:** Concise structural classes of representative MPPs, expected microbial utilization patterns, and key references.

MPP Class and Examples	Structural Determinants	Microbiota–Metabolite–Immune Implications	Evidence Interpretation and Key References
β-glucans/fungal polysaccharides: *Ganoderma lucidum*, *Poria cocos*, lentinan	β-(1→3)-D-glucan backbone, β-(1→6) branching, molecular-weight range, and triple-helix conformation differ by source/extraction.	β-glucan-degrading CAZyme modules can support *Bacteroides* and cross-feeding; reported relevance includes SCFA output, macrophage/Dectin-1 signaling, barrier effects, and antitumor immunity.	Promising but class-specific; fungal β-glucans should not be generalized to all MPPs. Key refs: [22,23,24,25,36].
Arabinogalactans: APS, LBP, *Angelica* polysaccharides	Galactan domains with arabinose-rich side chains; Ara/Gal ratio, branching degree, and molecular size are critical.	Debranching GH43/GH51 enzymes and *Bacteroides* PULs can coordinate utilization; preclinical evidence links these fractions to butyrate-supporting cross-feeding, Treg/Th17 balance, and macrophage modulation.	High mechanistic plausibility, but aging-specific confirmation remains limited. Key refs: [26,27,51,52,53].
Pectic/RG-I polysaccharides: *Panax ginseng*, yam and related plant pectins	Galacturonic-acid-rich homogalacturonan/RG domains; uronic acid content, esterification, acetylation, and side-chain architecture shape fermentation.	Pectate lyases, carbohydrate esterases, and uronic-acid-utilizing taxa such as *Bacteroides* may support SCFA production, bile-acid modulation, barrier reinforcement, and macrophage/T-cell effects.	Requires detailed uronic acid, esterification, and linkage reporting for reproducibility. Key refs: [29,30,41,54].
Glucomannans/galactomannans: konjac, guar gum	β-(1→4)-linked mannose/glucose or mannan backbones with galactose substitutions; viscosity and molecular weight are important translational variables.	Mannanase-associated taxa and GH5/GH9 enzymes can generate fermentable intermediates that support *Roseburia* and other butyrate producers.	Useful standardized comparators for MPP claims and head-to-head trials. Key refs: [31,32,37,45,47].
Polysaccharide–protein or conjugated complexes	Carbohydrate chains associated with protein/peptide or phenolic moieties; non-carbohydrate components may alter PRR binding and microbial catabolism.	Combined carbohydrate and nitrogen/peptide metabolism may broaden immunological effects but makes attribution of active moieties harder.	Purity, endotoxin, protein, and phenolic contents must be quantified. Key refs: [33,44].
Processed or chemically modified MPPs	Sulfation, acetylation, methyl esterification, honey processing, and partial hydrolysis alter charge, solubility, conformation, and enzymatic accessibility.	Modified substrates may require sulfatases/esterases or different CAZyme modules and can shift SCFA profile, barrier effects, and receptor signaling.	Promising for optimization, but modification-specific safety and batch control are essential. Key refs: [42,43,53].

## 4. Microbiota-Mediated Metabolite Reprogramming Induced by MPPs

### 4.1. SCFA Production and Immunoregulatory Functions

MPPs are structurally heterogeneous macromolecular carbohydrates whose fermentation behavior is largely determined by monosaccharide composition, glycosidic linkage patterns, molecular-weight distribution, branching architecture, and higher-order conformation. These structural features influence microbial substrate accessibility and thereby shape both the total yield and relative composition of SCFAs generated during microbial fermentation. Representative medicinal-source polysaccharides with distinct structures have been shown to produce different SCFA profiles in vitro and in vivo. For example, the exopolysaccharide EPS-LM isolated from the mycelia of Cordyceps militaris and the *Lycium barbarum* polysaccharide LBP can both be utilized as carbon sources by human fecal microbiota, but they differ substantially in monosaccharide consumption patterns and in the production of specific SCFAs, particularly propionate and butyrate [52]. Similarly, Chinese yam and its bioactive components, including mucilage polysaccharides and diosgenin, increase acetate and butyrate production during anaerobic fermentation [54]. Distinct fractions of *Dendrobium huoshanense*, including aqueous extract, polysaccharide fraction, and non-polysaccharide fraction, also differentially promote acetate, propionate, and butyrate production [55].

Structural modification can further alter microbial fermentation outcomes. Compared with the crude Astragalus polysaccharide APS2a, honey-processed Astragalus polysaccharide HAPS2a exhibits altered structural characteristics and induces higher levels of total SCFAs and other organic acids after in vitro fermentation [53]. These findings indicate that the fine structure of MPPs is a key determinant of selective microbial utilization and metabolite output, supporting the concept that structure-defined polysaccharides may generate distinct immunometabolic signatures.

SCFAs, particularly butyrate, are major molecular mediators linking microbial carbohydrate fermentation to host immune regulation. Their immunological effects are mediated mainly through two complementary mechanisms: activation of G-protein-coupled receptors (GPCRs) and inhibition of histone deacetylases (HDACs). Acetate, propionate, and butyrate function as endogenous ligands for GPR41, GPR43, and GPR109A [56]. For instance, polysaccharides from *Gynostemma pentaphyllum* have been reported to regulate oxidative stress responses under acute hypobaric hypoxia partly through GPR41 and GPR109A activation [57]. Through these receptors, SCFAs modulate the differentiation and function of intestinal immune cells. Butyrate and propionate promote the differentiation and suppressive activity of regulatory T cells (Treg cells) in the intestinal lamina propria, thereby maintaining mucosal tolerance and limiting excessive inflammation [58]. SCFAs also regulate macrophage polarization by suppressing pro-inflammatory M1 activation and promoting anti-inflammatory M2 phenotypes, which contributes to resolution of tissue inflammation [59].

Beyond immune cell regulation, SCFAs are essential for maintaining intestinal epithelial barrier integrity. Butyrate serves as a major energy substrate for colonocytes and enhances the expression of tight junction proteins, including zonula occludens-1 (ZO-1), occludin, and claudin-1, thereby reducing epithelial permeability and limiting microbial and endotoxin translocation [60,61]. As HDAC inhibitors, SCFAs increase histone acetylation and regulate the transcription of immune-related genes. Butyrate has also been reported to suppress pro-inflammatory SASP mediators, including IL-6 and IL-8, in senescent T cells, potentially through attenuation of mammalian target of rapamycin (mTOR) activation and nuclear factor-κB (NF-κB) signaling [58]. Thus, SCFA production represents a central mechanism through which MPPs may modulate Treg differentiation, macrophage polarization, epithelial barrier function, and inflammaging-associated immune dysfunction (Table 2).

### 4.2. Regulation of Secondary Bile Acid Metabolism

MPPs can modulate bile acid (BA) metabolism by reshaping the gut microbiota, particularly taxa with bile salt hydrolase (BSH) activity and bacteria capable of downstream BA transformation. Primary BAs are synthesized from cholesterol in the liver, conjugated, secreted into the intestine, and subsequently converted by gut microorganisms into SBAs through deconjugation, dehydroxylation, oxidation, epimerization, and related reactions [88]. Several polysaccharides have been reported to alter this microbial conversion process. Red kidney bean polysaccharides modulate BSH activity by enriching *Akkermansia*, thereby altering BA composition, especially taurocholic acid metabolism [89]. Mulberry leaf polysaccharides have been reported to increase the abundance of *Prevotella*, *Ruminococcus*, and *Lactobacillus*, contributing to the correction of BA metabolic disturbances [90]. Kelp polysaccharides enrich bacterial taxa such as Bacteroidia, Campylobacteria, and Clostridia and promote the production of SBAs, including nor-deoxycholic acid, taurolithocholic acid, and β-ursodeoxycholic acid [91]. Ginseng polysaccharides (GPSs) have likewise been shown to improve BA metabolic disorders [92]. Together, these findings suggest that polysaccharides can reshape the BA pool by selectively regulating microbial taxa involved in BA deconjugation and transformation.

SBAs are not merely digestive metabolites; they are signaling molecules that regulate metabolic and immune homeostasis. Lithocholic acid (LCA), deoxycholic acid (DCA), and related SBAs act through multiple receptors, most prominently farnesoid X receptor (FXR) and Takeda G-protein-coupled receptor 5 (TGR5). FXR activation suppresses hepatic BA synthesis and regulates lipid and glucose metabolism. Importantly, FXR and TGR5 signaling also modulate inflammatory responses. Activation of these receptors can inhibit NF-κB signaling and NLRP3 inflammasome activation, thereby reducing low-grade hepatic and systemic inflammation [93]. In metabolic dysfunction-associated steatotic liver disease, BA signaling through FXR and TGR5 has been implicated in the regulation of inflammatory and metabolic injury. In aging, gut dysbiosis-associated alterations in the SBA pool are closely linked to inflammaging [94]. Increased abundance of SBA-producing bacteria, including members of Ruminococcaceae, Lachnospiraceae, and *Clostridium*, may elevate DCA and related metabolites, which can aggravate neuroinflammation and cognitive decline through mechanisms involving microglial activation [95]. Conversely, BA profiles observed in some long-lived populations suggest that balanced BA metabolism may contribute to healthy aging [88].

Therefore, by regulating microbial BA transformation and optimizing SBA composition, MPPs may fine-tune the FXR/TGR5 signaling axis and related nuclear receptor networks. This provides a plausible mechanism by which MPPs could attenuate excessive inflammation, improve metabolic homeostasis, and modulate immune-aging processes [89,96]. However, most current evidence remains indirect, and future studies should establish causal links among defined polysaccharide structures, BA-transforming microbial taxa, specific SBA species, receptor activation, and immune-aging endpoints (Table 3).

### 4.3. Reorientation of Tryptophan Metabolism Toward Indole Derivatives

MPPs may also regulate host immunity by reshaping tryptophan metabolism. In aging and chronic inflammation, tryptophan metabolism often shifts toward the host-dominant kynurenine pathway, which is associated with increased indoleamine 2,3-dioxygenase activity, immune suppression, oxidative stress, and inflammatory signaling. In contrast, a microbiota-favorable metabolic state can enhance the production of indole derivatives with mucosal-protective and immunoregulatory properties. A wheat bran-rich diet, as a source of dietary fiber and polysaccharides, has been shown to suppress tryptophan conversion into kynurenine-pathway metabolites while increasing microbiota-derived indole metabolites, including indole-3-propionic acid (IPA), indole-3-acetaldehyde, and 5-hydroxyindole-3-acetic acid [130]. This shift is associated with enrichment of beneficial taxa such as *Akkermansia* and *Lactobacillus*. Although not a polysaccharide, astaxanthin has similarly been reported to increase indole-3-lactic acid (ILA) and IPA through gut microbiota modulation, supporting the broader concept that dietary bioactives can redirect tryptophan metabolism through microbial ecological remodeling [131].

Mechanistically, polysaccharide substrates may support indole-producing bacteria and influence microbial cross-feeding networks, thereby shifting tryptophan metabolic flux away from pro-inflammatory kynurenine-dominant patterns and toward microbiota-derived indole pathways. However, direct evidence linking structurally defined MPPs to specific indole-producing taxa and immune-aging outcomes remains limited and requires further causal validation.

Microbiota-derived indole derivatives, including ILA, IPA, indole-3-acetic acid (IAA), and indole-3-aldehyde (IAld), are endogenous ligands of the aryl hydrocarbon receptor (AhR) [132]. Through AhR activation, these metabolites exert multilayered effects on mucosal and systemic immunity. First, AhR signaling supports intestinal epithelial barrier integrity. ILA and IAA can upregulate tight junction proteins, including ZO-1 and occludin, in intestinal epithelial cells through AhR-dependent mechanisms, thereby reducing epithelial permeability [133]. In dextran sulfate sodium-induced colitis, ILA and IAA have been shown to repair intestinal barrier disruption [134]. Second, AhR activation can interact with antioxidant pathways, including nuclear factor erythroid 2-related factor 2 (Nrf2), thereby enhancing cellular antioxidant defenses [135]. In a pyrrolizidine alkaloid-induced hepatic sinusoidal obstruction syndrome model, microbial tryptophan derivatives such as indole-3-acetaldehyde and IAA activated the AhR/Nrf2 axis and alleviated hepatic oxidative stress and endothelial injury [135]. Third, indole–AhR signaling contributes to the regulation of T helper 17/regulatory T cell balance. AhR activation can promote Treg differentiation and function while restraining excessive Th17 activation in a context-dependent manner [136].

**Figure 3:**
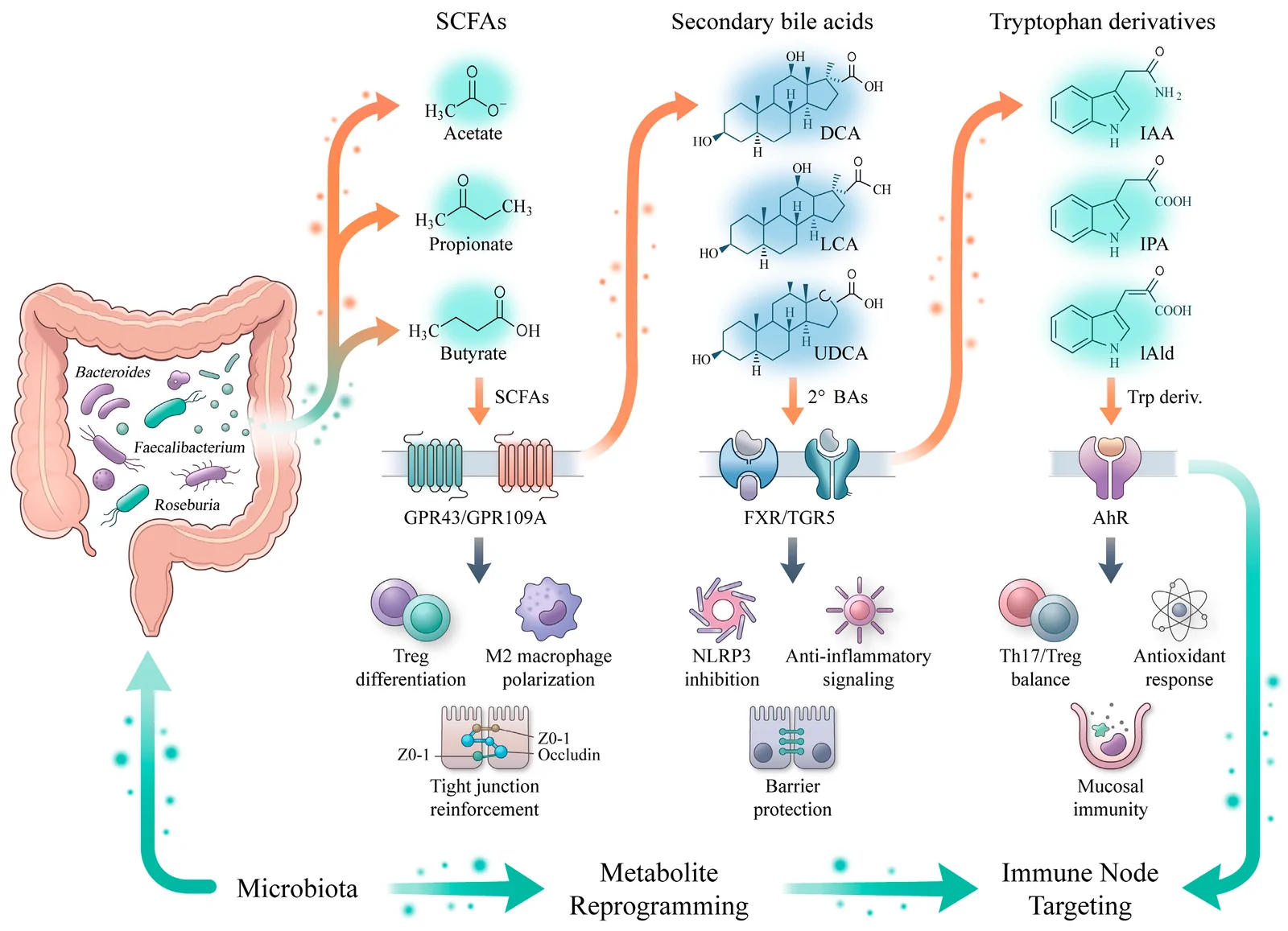
Proposed pathways of microbiota-mediated metabolite reprogramming associated with MPPs. Gut commensal bacteria may ferment MPPs into three major classes of immunoregulatory metabolites: SCFAs, including acetate, propionate, and butyrate; SBAs, including deoxycholic acid (DCA), lithocholic acid (LCA), and ursodeoxycholic acid (UDCA); and tryptophan-derived indoles, including IAA, IPA, and IAld. These metabolites may act through GPR43/GPR109A, FXR/TGR5, and AhR signaling pathways to regulate immune cell differentiation, inflammatory responses, intestinal barrier integrity, and mucosal immune homeostasis. This conceptual model integrates current evidence from MPPs and related dietary polysaccharide systems and should be interpreted as a putative framework rather than a fully validated pathway for any single MPP. Abbreviations: AhR, aryl hydrocarbon receptor; DCA, deoxycholic acid; FXR, farnesoid X receptor; GPR, G-protein-coupled receptor; IAA, indole-3-acetic acid; IAld, indole-3-aldehyde; IPA, indole-3-propionic acid; MPPs, medicinal plant polysaccharides; SBAs, secondary bile acids; SCFAs, short-chain fatty acids; TGR5, Takeda G-protein-coupled receptor 5; UDCA, ursodeoxycholic acid. This schematic was created by the authors using Adobe Illustrator 2026 (Adobe Inc., San Jose, CA, USA); no panel, icon arrangement, or graphical composition was reproduced or adapted from a published source.

Collectively, MPP-associated reprogramming of tryptophan metabolism may establish a protective immunometabolic network involving barrier reinforcement, oxidative stress control, and adaptive immune regulation. Nevertheless, given the context-dependent nature of AhR signaling, future studies should distinguish beneficial microbial indoles from host-derived kynurenine metabolites and determine whether specific MPP structures reproducibly promote protective indole signatures in aged hosts (Figure 3, Table 4).

## 5. Core Pathological Features of Immunosenescence and Inflammaging

### 5.1. Adaptive Immune Aging

Adaptive immune aging is a central component of immunosenescence and contributes to increased infection susceptibility, impaired vaccine responsiveness, and persistent inflammatory activation in older individuals [158]. This process affects both T-cell and B-cell compartments and is characterized by reduced immune reserve, restricted antigen-recognition capacity, and impaired immune homeostasis [159].

In the T-cell compartment, thymic involution is one of the earliest and most prominent age-associated alterations. Because the thymus is the primary site for naïve T-cell development, age-related reductions in thymic volume and cellularity markedly decrease the output of recent thymic emigrants [160,161]. This decline progressively depletes the peripheral naïve T-cell pool and contracts TCR diversity, thereby limiting the ability of the aged immune system to recognize novel antigens [162]. Such repertoire restriction is one mechanistic basis for the weakened responses of older adults to emerging pathogens, including severe acute respiratory syndrome coronavirus 2 (SARS-CoV-2) [163].

The loss of naïve T cells is accompanied by the expansion of memory and terminally differentiated effector memory T-cell subsets [160,164]. These cells frequently acquire senescent or exhausted phenotypes, including loss of CD28 and CD27, increased expression of CD57 and killer cell lectin-like receptor G1 (KLRG1), reduced proliferative potential, and impaired cytokine-producing capacity [165,166]. In parallel, exhausted T cells often express inhibitory receptors such as programmed cell death protein 1 (PD-1) and T-cell immunoglobulin and mucin-domain-containing protein 3 (Tim-3) [165,167]. Beyond functional impairment, senescent and exhausted T cells can actively amplify tissue inflammation through SASP-associated mediators, thereby contributing to inflammaging and age-related tissue dysfunction [159,164].

B-cell aging similarly compromises humoral immunity. Aging reduces B-cell generation in the bone marrow and secondary lymphoid organs, leading to contraction of the naïve B-cell compartment [162,168]. This is accompanied by the accumulation of antigen-experienced B-cell subsets, particularly age-associated B cells (ABCs), which typically display a CD21− CD23− phenotype and may express T-box transcription factor TBX21 (T-bet) and CD11c [169]. B-cell receptor (BCR) diversity also declines, limiting the generation of high-affinity antigen-specific antibodies [162]. Together with impaired germinal center reactions and defective T–B-cell cooperation, these changes weaken antibody responses to novel antigens and contribute to poor vaccine efficacy in older adults [170]. Moreover, dysregulated immune tolerance in aging facilitates the escape of autoreactive B-cell clones and enhances autoantibody production, linking B-cell immunosenescence to increased autoimmune risk [162,171].

Collectively, adaptive immune aging is defined by diminished naïve lymphocyte output, reduced receptor diversity, accumulation of senescent or exhausted lymphocytes, impaired vaccine responsiveness, and increased inflammatory and autoimmune propensity. These features provide key immune endpoints for evaluating microbiota-directed interventions targeting immunosenescence (Table 5).

### 5.2. Innate Immune Dysregulation and the Inflammaging Circuit

Innate immune aging is another major driver of inflammaging. In aged hosts, macrophages and dendritic cells exhibit altered pattern-recognition receptor (PRR) signaling, impaired phagocytic clearance, and exaggerated inflammatory responses to microbial products or damage-associated signals. Aberrant activation of the NLRP3 inflammasome is particularly relevant because it links gut microbiome-derived inflammatory cues to vascular inflammation and age-related diseases such as atherosclerosis [172].

Aged macrophages show reduced capacity to clear apoptotic cells and pathogens, thereby promoting persistence of inflammatory stimuli within tissue microenvironments. At the same time, pro-inflammatory macrophage polarization is enhanced, leading to increased secretion of IL-1β, IL-6, TNF-α, and monocyte chemoattractant protein-1 (MCP-1) [173]. Barrier dysfunction further reinforces this process. For example, in chronic heart failure, translocation of bacterial products caused by impaired intestinal barrier integrity continuously activates innate immune pathways and is associated with worsening cardiac dysfunction [174]. These findings support the view that innate immune dysregulation is not merely a consequence of aging but an active amplifier of systemic inflammation.

The SASP further sustains this inflammatory circuit. Senescent parenchymal and immune cells release inflammatory cytokines, chemokines, growth factors, and proteases that act locally and systemically. In inflammatory bowel disease, neutrophils and other innate immune cells can undergo trained-immunity-like reprogramming, generating long-lasting pro-inflammatory memory even after the initial stimulus has resolved [175]. SASP-associated mediators, including IL-6 and TNF-α, can also activate tissue-resident macrophages such as microglia, thereby contributing to neuroinflammation and neurodegenerative pathology, including Alzheimer’s disease [176].

Barrier impairment is a critical interface between the SASP and microbiota-driven inflammation. Aging-associated gut dysbiosis and altered microbial metabolite profiles can damage mucosal barrier integrity and induce immunometabolic dysfunction [177]. This permits increased translocation of lipopolysaccharide (LPS) and other microbial products into the circulation. LPS activates Toll-like receptor 4 (TLR4) and other PRRs, further stimulating NF-κB signaling, NLRP3 inflammasome activation, and IL-1β production [178]. A self-amplifying loop is therefore established: senescent immune and tissue cells secrete SASP factors; barrier integrity declines; microbial products enter the circulation; innate immune pathways are aberrantly activated; and additional inflammatory mediators are produced. This inflammaging circuit provides a common pathological basis for cardiovascular disease, metabolic dysfunction-associated steatotic liver disease, and neurodegenerative disorders [179,180].

Mechanistically, NF-κB and NLRP3 inflammasome signaling form a two-step inflammatory module that is highly relevant to microbiota-driven inflammaging. Microbial products such as LPS provide a priming signal through TLR4–MyD88-dependent NF-κB activation, increasing transcription of NLRP3, pro-IL-1β, pro-IL-18, IL-6, and TNF-α. A second activation signal, including mitochondrial reactive oxygen species, potassium efflux, lysosomal damage, or extracellular ATP, promotes NLRP3 assembly, caspase-1 activation, and maturation of IL-1β and IL-18. SCFAs and indole derivatives may restrain this circuit by strengthening epithelial barrier integrity, inhibiting HDAC-dependent inflammatory transcription, activating GPR43/GPR109A or AhR signaling, and reducing oxidative-stress-driven inflammasome activation [132,133,134,135,178]. This provides a mechanistic bridge between MPP fermentation, metabolite production, and suppression of innate immune inflammaging.

Interrupting this circuit is therefore a central goal of microbiota-directed interventions. In the context of this review, SCFAs, SBAs, and tryptophan-derived indoles are particularly relevant because they can regulate macrophage polarization, inflammasome activity, epithelial barrier integrity, and SASP-associated inflammatory signaling.

### 5.3. Mucosal Immune Aging and the Gut–Immune Axis

The intestinal mucosal immune system is highly sensitive to aging and constitutes a major interface between microbial dysbiosis and systemic immunosenescence. Age-associated mucosal immune decline is characterized by impaired epithelial barrier integrity, reduced Paneth cell function, altered gut-associated lymphoid tissue (GALT) activity, and dysregulated communication between intestinal and systemic immune compartments.

The intestinal epithelial barrier depends on coordinated interactions among epithelial cells, tight junction proteins, mucus, antimicrobial peptides, and mucosal immune cells. With aging, barrier integrity declines, leading to increased intestinal permeability, commonly referred to as “leaky gut” [181]. This facilitates the systemic translocation of LPS and other microbial products, thereby promoting chronic low-grade inflammation [182]. Importantly, barrier dysfunction and gut dysbiosis reinforce one another. In aged mouse models, intestinal barrier disruption occurs together with altered microbial composition, whereas FMT from young donors improves barrier integrity in aged recipients, supporting a causal role of microbiota in age-related intestinal homeostatic failure [183].

Paneth cell dysfunction further weakens mucosal defense. Paneth cells, located at the base of small-intestinal crypts, secrete antimicrobial peptides such as lysozyme and defensins, which help maintain microbial spatial organization and protect against opportunistic pathogens. Aging reduces Paneth cell antimicrobial output, thereby weakening innate intestinal defense and increasing susceptibility to pathogen colonization and infection [184].

GALT also undergoes age-related structural and functional decline. GALT includes Peyer’s patches, isolated lymphoid follicles, and mesenteric lymph nodes and serves as a major site for antigen sampling, immune tolerance, and antigen-specific immune activation [185]. Aging alters immune cell composition and antigen-presenting capacity within the intestinal mucosa, reduces T-cell and B-cell responsiveness, and may disturb Treg-cell homeostasis [184]. Although certain tissue-resident memory T cells can maintain relatively stable phenotypes during aging, circulating effector memory T cells increasingly express senescence-associated markers and display functional exhaustion [186]. Thus, mucosal immunosenescence is not confined to the intestine but can influence systemic immune aging through the gut–immune axis.

The gut–immune axis provides a mechanistic rationale for targeting the microbiota to delay immunosenescence. The intestine is both a digestive and immune organ, and its mucosal immune network is closely integrated with systemic immunity [187]. Age-related barrier disruption allows gut-derived inflammatory signals to activate circulating immune cells, promote senescent immune phenotypes, and accelerate thymic involution [188]. Reduced naïve T-cell output then forces peripheral T-cell maintenance to rely more heavily on clonal expansion of memory subsets, further aggravating replicative senescence and reducing immune diversity [188].

Microbiota-directed interventions can partially counteract this process. Dietary prebiotics, probiotics, FMT, and microbial metabolites such as SCFAs can improve barrier function, regulate mucosal immune responses, and reduce systemic inflammatory tone [94]. For example, metformin remodels the aged gut microbiota, increases beneficial taxa such as *Akkermansia*, and is associated with reduced splenic immunosenescence, including lower pro-inflammatory cytokine levels and improved cytotoxic T-cell profiles [189]. Similarly, Bacillus DS2 produces indole-3-lactic acid, activates AhR signaling, strengthens mucosal immunity, and alleviates inflammaging in aged mice [190]. These findings support the concept that the gut–immune axis is a tractable target for microbiota-directed strategies aimed at mitigating immune aging.

## 6. Microbial Metabolites as Functional Mediators Targeting Immunosenescence Nodes

### 6.1. Restoration of T-Cell Homeostasis

Microbiota-derived metabolites, particularly SCFAs and indole derivatives, provide a mechanistic link between polysaccharide fermentation and adaptive immune remodeling. SCFAs regulate T-cell fate partly through inhibition of histone deacetylases (HDACs), thereby promoting Treg differentiation and restraining excessive pro-inflammatory Th17 responses [191]. Butyrate, in particular, downregulates retinoic acid receptor-related orphan receptor γt (RORγt) and IL-17 while increasing forkhead box P3 (Foxp3) expression, thereby favoring restoration of the Th17/Treg balance [192]. In experimental ulcerative colitis, APS restored SCFA production through a microbiota-dependent mechanism and improved Th17/Treg imbalance in parallel with inhibition of NF-κB activation [193]. Similarly, litchi procyanidins promoted colonic Treg expansion through GPR43 signaling, supporting the broader relevance of SCFA receptor-mediated immune regulation [194].

SCFAs also influence T-cell metabolism and effector function through GPR43 and GPR109A. In allogeneic hematopoietic stem cell transplantation models, SCFA signaling maintained metabolic homeostasis in alloreactive T cells [195]. In cancer models, SCFAs enhanced the antitumor activity of cytotoxic T lymphocytes and chimeric antigen receptor T (CAR-T) cells, suggesting that microbial metabolites can modulate both tolerogenic and effector T-cell responses depending on the immunological context [196]. In aging, dysbiosis-associated alterations in the SCFA pool may aggravate mucosal barrier dysfunction, immunometabolic imbalance, and T-cell senescence [177].

Indole derivatives generated from microbial tryptophan metabolism further regulate T-cell homeostasis through AhR signaling. Indole-3-acetic acid and related metabolites act as AhR ligands and participate in the control of intestinal Th17/Treg balance and immune cascades under chronic inflammatory conditions, including human immunodeficiency virus (HIV) infection [197]. Experimental models of nanoparticle exposure further indicate that reduced indole-3-acetic acid levels weaken AhR-mediated immune regulation [198]. Together, SCFAs and indole derivatives form a complementary metabolic network: SCFAs primarily act through HDAC inhibition and GPR-dependent signaling, whereas indole derivatives act mainly through AhR. This network regulates T-cell activation, proliferation, differentiation, and inflammatory polarization, thereby providing a plausible mechanism through which MPP-driven microbial metabolism may mitigate T-cell immunosenescence.

### 6.2. Regulation of Innate Immune Cell Dysfunction

Innate immune dysregulation is a major driver of inflammaging, and microbial metabolites are key regulators of macrophage and dendritic cell function. SCFAs and SBAs can directly modulate the inflammatory state of innate immune cells [177]. A central mechanism is suppression of NLRP3 inflammasome activation, which reduces maturation and release of IL-1β and IL-18, two cytokines closely linked to chronic inflammatory amplification during aging [177]. These metabolites also influence antigen presentation and cytokine secretion by dendritic cells, thereby indirectly shaping downstream adaptive immune responses [199].

SCFAs, particularly butyrate and propionate, promote macrophage polarization toward an anti-inflammatory and tissue-repair phenotype. This shift enhances apoptotic cell clearance, supports tissue remodeling, and limits excessive inflammatory signaling [177]. SCFAs may also improve the phagocytic clearance of senescent cells, thereby reducing SASP-associated inflammatory burden at its source [177]. Under inflammatory conditions, acetate and propionate can further downregulate TNF-α and IL-8 expression in intestinal epithelial and immune cells while modulating tight junction proteins, thereby integrating anti-inflammatory and barrier-protective effects [200].

Thus, the innate immune effects of microbial metabolites converge on three major immunosenescence-related nodes: inhibition of inflammasome activation, restoration of macrophage functional plasticity, and reinforcement of epithelial–immune barrier coordination. These actions are particularly relevant to MPPs because polysaccharide fermentation can alter the availability of SCFAs, SBAs, and indole derivatives in the intestinal microenvironment.

### 6.3. Maintenance of Barrier Integrity and Immune Tolerance

The intestinal barrier is a critical interface between microbial metabolism and systemic immune aging. Among microbial metabolites, butyrate is especially important because it serves as a preferred energy source for colonocytes and strengthens epithelial barrier integrity. It upregulates tight junction proteins, including claudin-1, claudin-5, and zonula occludens-1 (ZO-1). Long-term intake of Camellia polysaccharides increased colonic mRNA expression of these barrier-related proteins [201], whereas Trichosanthes polysaccharides restored epithelial barrier integrity in diabetic models [202]. Butyrate also promotes mucin 2 (MUC2) expression and mucus secretion, thereby reinforcing the chemical barrier and limiting microbial translocation [203].

Microbial metabolites also contribute to immune tolerance by activating host receptor pathways. AhR responds to tryptophan-derived metabolites such as indole-3-propionic acid (IPA), thereby regulating mucosal immune cell function and intestinal tolerance [204]. In sepsis-associated liver injury, reduced IPA levels are associated with weakened anti-inflammatory signaling [205]. SBAs can promote peripherally derived Treg differentiation through farnesoid X receptor (FXR)-dependent signaling, whereas SCFAs induce Treg differentiation through both HDAC inhibition and activation of GPR43/GPR109A [206]. GPR43 and GPR109A are expressed in barrier-associated cell populations and contribute to tissue barrier maintenance [207].

**Figure 4:**
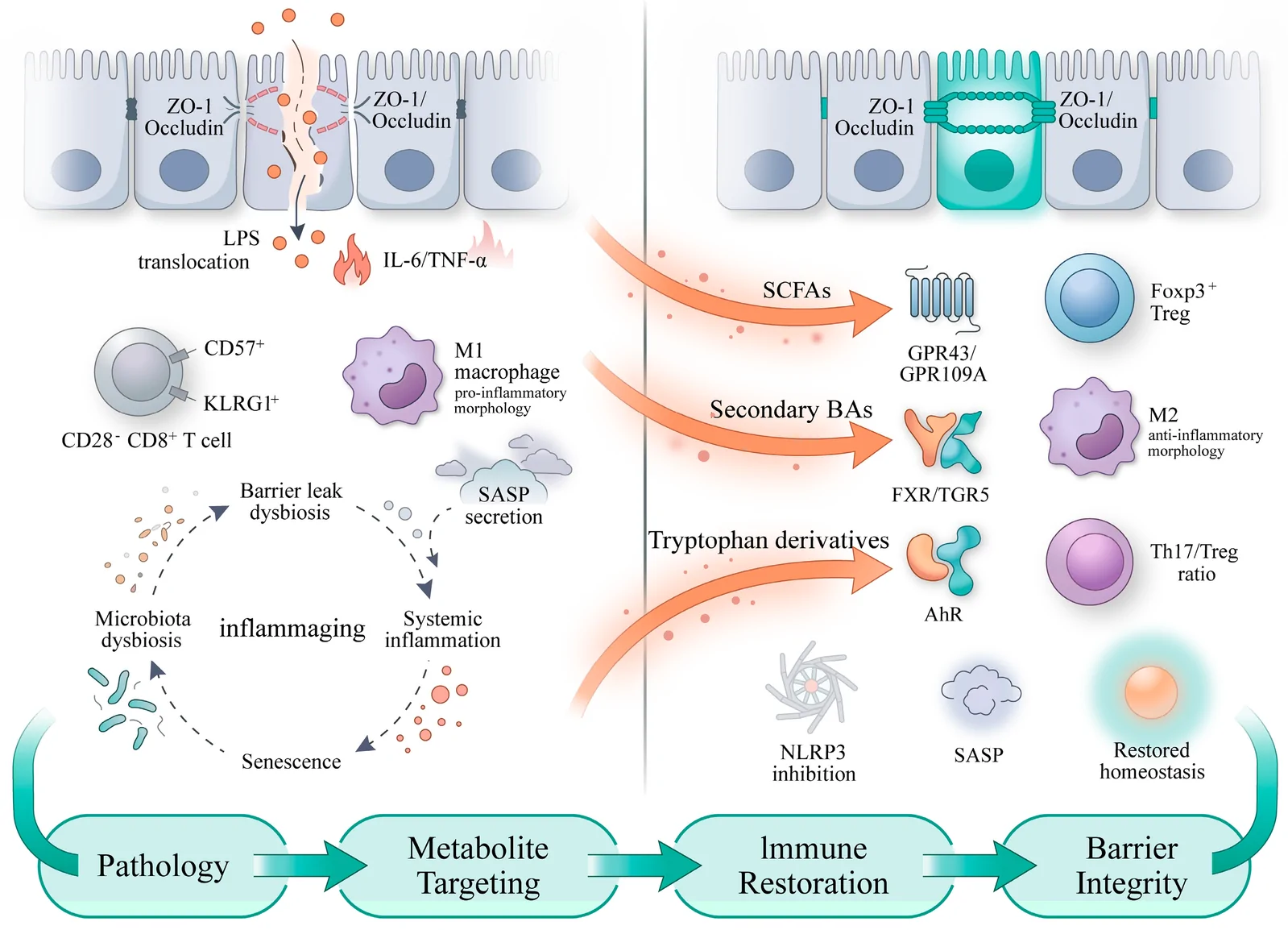
Proposed metabolite-targeted regulation of immunosenescence and inflammaging. The left panel illustrates the pathological cycle of immunosenescence: age-associated gut dysbiosis impairs epithelial barrier integrity, promotes LPS translocation, and amplifies systemic inflammaging. This inflammatory environment accelerates immune cell senescence, including expansion of CD28− CD57+ KLRG1+ CD8+ T cells, pro-inflammatory macrophage polarization, and SASP secretion, which further aggravates barrier dysfunction and dysbiosis. The right panel summarizes putative mechanisms by which MPP-associated microbial metabolites may contribute to immune homeostasis. SCFAs, SBAs, and tryptophan-derived indoles may activate receptor pathways including GPR43/GPR109A, FXR/TGR5, and AhR, thereby promoting Foxp3^+^ Treg-cell differentiation, supporting Th17/Treg-cell balance, favoring M2-like macrophage polarization, inhibiting NLRP3 inflammasome activation, reducing SASP-associated inflammation, and strengthening epithelial barrier integrity. This figure represents an integrated conceptual model rather than a pathway validated for a single MPP preparation. Abbreviations: AhR, aryl hydrocarbon receptor; FXR, farnesoid X receptor; GPR, G-protein-coupled receptor; KLRG1, killer cell lectin-like receptor G1; LPS, lipopolysaccharide; MPPs, medicinal plant polysaccharides; NLRP3, NOD-like receptor family pyrin domain-containing 3; SASP, senescence-associated secretory phenotype; SBAs, secondary bile acids; SCFAs, short-chain fatty acids; TGR5, Takeda G-protein-coupled receptor 5; Th17, T helper 17 cell; Treg, regulatory T cell; CD, cluster of differentiation; Foxp3, forkhead box P3. This schematic was created by the authors using Adobe Illustrator 2026 (Adobe Inc., San Jose, CA, USA); no panel, icon arrangement, or graphical composition was reproduced or adapted from a published source.

Collectively, SCFAs, SBAs, and indole derivatives act on intestinal epithelial cells, dendritic cells, macrophages, and T cells to establish a tolerogenic mucosal microenvironment. By reinforcing barrier integrity and limiting aberrant immune activation against commensal microorganisms, these metabolites interrupt the leaky gut–endotoxemia–inflammaging axis and provide a mechanistic basis for microbiota-directed interventions targeting immunosenescence (Figure 4) [94].

## 7. Preclinical and Clinical Evidence for MPPs in Immunosenescence Intervention

### 7.1. Evidence from Aging Animal Models

Preclinical studies provide the strongest current support for the potential of MPPs to modulate immunosenescence through the microbiota–metabolite axis. In D-galactose-induced accelerated aging models, several polysaccharide-containing interventions have improved immune, oxidative stress, and tissue-aging endpoints. UP360, a standardized mushroom-based composition containing polysaccharide-rich components from *Aloe vera*, *Poria cocos*, and *Rosmarinus officinalis*, enhanced innate and adaptive immune responses, increased superoxide dismutase (SOD) and nuclear factor erythroid 2-related factor 2 (Nrf2) levels, improved antioxidant capacity, and protected the thymus against aging-related injury [208]. White mushroom polysaccharides (WMPs) improved locomotor activity, spatial memory, and recognition memory while reducing oxidative stress and pro-inflammatory cytokine levels in the brain. These effects were associated with increased gut α-diversity, elevated SCFA levels, and enrichment of *Bacteroides* and Parabacteroides, suggesting microbiota-linked anti-aging activity [209].

Structure-focused studies further support the importance of polysaccharide heterogeneity. A green extraction strategy for LBP yielded rhamnogalacturonan-I-type pectin–protein complexes with distinct physicochemical and biological properties. Among these fractions, S1-A showed the strongest immunoenhancing activity, whereas S1-M1 most effectively promoted *Bacteroides* proliferation, providing evidence that defined structural fractions may differentially regulate immune and microbial endpoints [210].

Microbiota dependence has been tested more directly in depletion models. *Cistanche deserticola* polysaccharides (CDPSs) improved cognitive function and alleviated amino acid imbalance, peripheral inflammation, and oxidative stress in D-galactose-treated mice by restoring microbial homeostasis. However, these benefits were abolished after antibiotic-mediated microbiota ablation or immunosuppression, indicating that an intact microbiota and immune system are required for their activity [211]. *Dendrobium officinale* polysaccharides (DOPs) and Camellia flower polysaccharides (TFPSs) also remodeled the gut microbiota of aged mice, reduced the Firmicutes/Bacteroidetes ratio, increased *Lactobacillus* abundance, and attenuated oxidative injury in neuroglial cells and hippocampal microglial activation [212,213].

Overall, animal studies support the biological plausibility that MPPs can improve immunosenescence-related indices, reduce inflammatory and oxidative injury, and protect tissue function through microbiota-associated mechanisms. Nevertheless, most studies remain associative, and only a limited subset has used antibiotic depletion, germ-free models, FMT, receptor antagonism, or metabolite rescue experiments to validate causality. A broader comparison of representative MPPs, integrating their structural characteristics, experimental models, microbiota and metabolite changes, immune endpoints, evidence levels, and major limitations, is provided in Table S1. A structured summary of the preclinical and translational evidence is provided in Table 6.

### 7.2. Human Observational Evidence

Human observational studies provide indirect but clinically relevant evidence linking plant polysaccharide intake, gut microbiota function, and immune aging. Cross-sectional studies have identified differences in microbial composition, polysaccharide-degradation capacity, and immunometabolic profiles between healthy and frail older adults. In individuals older than 60 years, fecal *Bifidobacterium* abundance is often reduced, potentially reflecting insufficient intake of fermentable substrates, including plant polysaccharides, as well as age-related changes in the intestinal environment [214]. Baseline microbial structure also influences responsiveness to polysaccharide-like substrates. In older adults, the relative abundance of *Prevotella* stratifies individuals into response groups after arabinoxylan oligosaccharide supplementation, indicating that microbiome configuration can determine metabolic and microbial responses to dietary polysaccharides [221].

Frailty is commonly associated with more severe dysbiosis, including reduced α-diversity, enrichment of potentially pathogenic taxa, and lower SCFA availability. These alterations may weaken the production of immunoregulatory metabolites and reduce the capacity to restrain inflammaging and immune cell dysfunction [222,223]. Dietary patterns rich in complex plant-derived polysaccharides, such as the Mediterranean diet, are also associated with healthier aging trajectories, partly through modulation of the gut microbiota and microbial metabolism. LBPs and other representative MPPs have been reported to regulate microbial homeostasis, enhance immune function, and alleviate oxidative stress-related inflammation [224]. In long-lived populations, including centenarians, distinctive microbial configurations may reflect long-term dietary exposure to complex plant polysaccharides, which continuously support commensal metabolism and beneficial metabolite production [225,226].

These observational findings support the rationale for targeting microbial polysaccharide utilization to preserve immune function during aging. However, they should be interpreted as associative evidence rather than proof that specific MPPs directly delay immunosenescence in humans.

### 7.3. Preliminary Human Intervention Trials

Human intervention evidence remains limited but provides important translational signals. A double-blind randomized controlled trial showed that a non-digestible polysaccharide (NPS) formulation used as an oral adjuvant improved seroprotection after seasonal influenza vaccination in healthy adults aged 50–79 years and reduced the incidence of common cold [215]. Another study reported that galactooligosaccharide (GOS) supplementation increased *Bifidobacterium* counts in pre-frail and healthy older adults, suggesting partial correction of aging-associated dysbiosis [214]. Although these interventions are relevant to microbiota-directed immune modulation, they are not equivalent to structurally characterized MPPs and should therefore be regarded as supportive rather than direct evidence.

Trials involving polysaccharide-rich plant extracts have also generated preliminary positive signals. Lactose-hydrolyzed milk supplemented with mulberry leaf and corn silk extracts reduced postprandial glucose peaks and glycemic fluctuations in patients with type 2 diabetes mellitus, suggesting potential metabolic benefits that may involve microbial metabolic regulation [216]. A Pistacia atlantica resin ointment containing polysaccharide components improved joint stiffness, pain, and function in older patients with osteoarthritis compared with placebo [217]. However, because these interventions used complex extracts or topical formulations, attribution of clinical effects specifically to polysaccharide-mediated microbiota modulation remains uncertain.

Current clinical studies have several limitations: small sample sizes, short intervention durations, limited structural characterization of polysaccharide preparations, insufficient mechanistic profiling, and weak attribution of outcomes to polysaccharide-mediated microbiota remodeling [218,219,220]. To move MPPs from “promising but not clinically established” candidates to validated tools for immunosenescence, the field should require a staged evidence package: (i) a good manufacturing practice (GMP)-compatible, batch-reproducible MPP preparation with predefined structural specifications and contaminant testing; (ii) phase I tolerability and dose-finding data in older adults; (iii) at least two independent, adequately powered, double-blind, placebo-controlled randomized trials in middle-aged or older adults; (iv) prespecified immune-aging endpoints, such as naïve/memory T-cell distribution, CD28 loss, CD57/KLRG1 expression, Th17/Treg balance, vaccine-response metrics, inflammatory cytokines, and barrier biomarkers; (v) parallel metagenomic, metabolomic, and immune-phenotyping evidence showing that clinical benefit tracks with microbial degradation capacity and SCFA/SBA/indole remodeling; and (vi) durability and safety follow-up sufficient to assess sustained immune and metabolic effects. Until this standard is met, MPPs should be described as mechanistically promising microbiota-directed candidates rather than validated clinical interventions for immunosenescence.

## 8. Current Research Gaps and Challenges

### 8.1. Limited Mechanistic Resolution

Current research on MPPs as microbiota-directed modulators of immunosenescence remains largely dominated by correlative observations. A major unresolved issue is the lack of direct experimental validation for the complete causal chain linking a defined polysaccharide structure to specific microbial degraders, microbial metabolites, host receptors, and immune-aging endpoints. For example, extraction-dependent Lycium barbarum polysaccharide (LBP) fractions with distinct physicochemical properties show different biological activities: the S1-A fraction enhances phagocytic activity and nitric oxide release in RAW 264.7 macrophages, whereas the S1-M1 fraction most strongly promotes the proliferation of Bacteroides [210]. However, these findings mainly indicate in vitro immunostimulatory potential and taxon-level microbial selectivity. They do not yet establish whether specific LBP structural motifs are metabolized by defined bacterial strains into particular immunoregulatory metabolites that act on discrete immune cell subsets during aging. Similarly, in Hashimoto’s thyroiditis, increased abundance of Bacteroides fragilis and its putative immune-regulatory function through polysaccharide A have been reported, but direct causal validation remains insufficient [227].

This mechanistic fragmentation is not unique to MPPs. Evidence from adjacent polysaccharide systems also illustrates the same limitation. For instance, sulfated polysaccharides from sea cucumber viscera can increase the abundance of *Akkermansia* and *Lactobacillus* and promote SCFA production, but the molecular sequence from polysaccharide structure to beneficial microbial taxa, then to specific metabolites, and finally to immune targets remains incompletely defined [228]. For MPPs, this gap is particularly important because their structural heterogeneity makes it difficult to attribute biological outcomes to defined molecular features. Future studies should therefore move beyond broad associations between “polysaccharides,” “microbiota,” and “immunity” and adopt causal designs, including gnotobiotic models, strain-level microbial knockouts, isotope tracing, metabolite receptor antagonism, and immune cell-specific pathway inhibition.

Another major limitation is insufficient spatiotemporal resolution. Most studies measure fecal microbial composition or bulk metabolite concentrations, which provides limited insight into local metabolite gradients and immune effects within tissue microenvironments. Although polysaccharide digestion and fermentation can be viewed as a coordinated process driven by host and microbial enzymes, little is known about real-time fluctuations in metabolite concentrations within Peyer’s patches, mesenteric lymph nodes, the spleen, or intestinal lamina propria, or about how these fluctuations are coupled to aging-related dysfunction in dendritic cells, T cells, B cells, and macrophages [229,230,231]. Integrating mass spectrometry imaging, spatial metabolomics, single-cell immunophenotyping, and in vivo real-time monitoring will be essential for defining how MPP-derived microbial metabolites reshape immune-aging niches.

### 8.2. Structural Heterogeneity and Standardization

The translational potential of MPPs depends on reproducible product quality and well-defined structure–function relationships. However, intrinsic structural heterogeneity remains a central obstacle. This heterogeneity arises from multiple sources, including plant species, geographical origin, growth stage, cultivation conditions, harvested plant part, extraction solvent, precipitation method, purification procedure, and drying process. For example, ethanol- and acetone-precipitated polysaccharide fractions from Zygophyllum geslini differ in yield, pH, and IC50 values, whereas the accumulation of Astragalus polysaccharides (APS) is influenced by origin, growth age, cultivation mode, and plant part [232,233,234]. Such variability directly affects microbial accessibility, fermentation kinetics, metabolite output, and downstream immunological effects.

A minimum characterization package should be reported for every MPP study intended to support structure–function interpretation. This package should include: botanical or fungal source with an authenticated Latin binomial and plant/fungal part; extraction, deproteinization, decolorization, purification, and drying procedures; yield, purity, endotoxin, residual protein, phenolic, nucleic acid, ash, and moisture contents; monosaccharide composition by high-performance anion-exchange chromatography with pulsed amperometric detection (HPAEC–PAD) or gas chromatography–mass spectrometry (GC–MS) after validated hydrolysis; molecular-weight distribution and dispersity by high-performance size-exclusion chromatography/gel permeation chromatography (HPSEC/GPC), preferably coupled to multi-angle light scattering; glycosidic linkage and branching analysis by methylation–GC–MS and one- or two-dimensional nuclear magnetic resonance (NMR) spectroscopy; uronic acid content and degree of methyl esterification/acetylation; sulfate or phosphate content when present; higher-order conformation by Congo red assay, circular dichroism, atomic force microscopy, scanning/transmission electron microscopy, or rheology where relevant; and batch fingerprints by Fourier-transform infrared spectroscopy (FTIR), NMR, chromatography, or mass spectrometry [235,236,237]. Reporting only the crude extract name, total sugar content, and one average molecular weight is insufficient for reproducibility.

Future work should prioritize activity-linked quality control before advanced prediction tools. The immediate priority is to establish reference materials, validated analytical methods, and release specifications that connect structure to microbial utilization and immune outcomes. Only after these foundations are in place should multi-omics, artificial intelligence-assisted structure–activity modeling, and microbial metabolic network reconstruction be used to predict the ability of MPPs to enrich specific primary degraders, regulate CAZyme/PUL-dependent fermentation, and reprogram SCFAs, SBAs, and indole derivatives. This prioritization should improve cross-study comparability, reduce batch effects, and make clinical translation more credible.

### 8.3. Interindividual Microbiome Variability

The efficacy of MPPs depends heavily on metabolic transformation by the gut microbiota. However, baseline microbiome composition varies markedly among individuals, especially in older populations, and this variation directly influences the utilization and biological effects of polysaccharides. After exposure to the same dietary fiber or polysaccharide substrate, different individuals can produce distinct amounts and proportions of SCFAs, and the same polysaccharide may be metabolized by different dominant microbial taxa into divergent metabolite profiles [238,239,240]. For example, studies of arabinoxylan oligosaccharides have shown that the presence or absence of baseline *Prevotella* strongly determines the microbial response pattern [221]. This finding highlights the importance of baseline microbial ecology in shaping polysaccharide responsiveness.

Therefore, defining “responders” and “non-responders” has become a key challenge for precision MPP intervention. Response classification should not rely solely on changes in microbial abundance; instead, it should integrate clinical outcomes, immune-aging biomarkers, microbial functional genes, and metabolite profiles. Baseline functional features, particularly CAZyme repertoires and polysaccharide utilization capacity, may predict whether an individual can efficiently degrade and benefit from a given MPP. Metagenomics, metabolomics, and immune phenotyping can be integrated to construct predictive models that identify likely beneficiaries before intervention and guide the design of personalized or stratified polysaccharide formulations [241,242,243,244]. This shift from a “one-size-fits-all” model to microbiome-informed precision nutrition is essential for improving the reproducibility and clinical effectiveness of MPP-based interventions. To improve reproducibility and facilitate clinical translation, a minimum structural-characterization and quality-control checklist for future MPP studies is provided in Table S2.

## 9. Future Directions and Translational Opportunities

### 9.1. Precision Microbiota-Directed Nutritional Intervention

Future research should be prioritized in a stepwise sequence. Priority 1 is structural and manufacturing standardization: define reproducible MPP fractions and analytical release criteria before broad biological testing. Priority 2 is causal preclinical validation in naturally aged or accelerated-aging models using microbiota depletion, FMT, gnotobiotic systems, isotope tracing, receptor antagonism, and metabolite rescue experiments. Priority 3 is responder stratification based on baseline CAZyme repertoires, PUL abundance, primary-degrader ecology, and metabolite-producing capacity. Priority 4 is clinical validation in older adults using standardized preparations, prespecified immune-aging endpoints, and integrated metagenomics, metabolomics, barrier markers, and immune phenotyping. Advanced multi-omics and artificial intelligence tools are valuable, but they should serve these priorities rather than replace rigorous product definition and causal testing.

Metagenomic profiling can be used to evaluate the abundance of functional genes involved in polysaccharide degradation, thereby enabling selection of the most suitable polysaccharide for a given individual or population subgroup [245]. In parallel, next-generation probiotics containing defined polysaccharide-degrading strains, or synbiotic formulations combining MPPs with compatible probiotics, should be developed to enhance targeted efficacy. In such systems, polysaccharides may serve as selective carbon sources that support probiotic colonization, while probiotics further degrade polysaccharides to optimize metabolite output [246]. For example, a composite hydrogel containing *Morinda officinalis* polysaccharides and inulin reshapes the microbiota through synergistic fermentation, increases propionate and butyrate levels, and activates peroxisome proliferator-activated receptor-γ (PPARγ) signaling, with superior effects compared with free polysaccharides [247]. In the future, highly efficient degrading strains may be screened and delivered together with MPPs using nanoparticles, hydrogels, or other colon-targeted systems to improve mucosal retention, microbial utilization, metabolic reprogramming, and immunosenescence-related outcomes [247,248].

### 9.2. Biomarker Development and Clinical Trial Design

A multidimensional biomarker framework is needed to evaluate the immunogerontological effects of MPPs. Such a framework should integrate gut microbial functional capacity, metabolite reprogramming, and immune-aging phenotypes. Microbial readouts may include CAZyme abundance, polysaccharide utilization loci, strain-level changes in primary degraders, and cross-feeding networks. Metabolic readouts should include SCFAs, SBAs, indole derivatives, and tryptophan metabolites. Immune-aging readouts should include CD57 and KLRG1 expression, CD28 loss, naïve T-cell frequency, Th17/Treg balance, macrophage polarization, inflammatory cytokines, and DNA methylation-based biological age clocks [249,250,251,252]. Combining these parameters would allow a more precise evaluation of how MPPs influence the microbiota–metabolite–immunity axis during aging.

Future clinical studies should move from short-term exploratory trials toward mechanistically integrated randomized controlled trials (RCTs). Recommended designs include a run-in period for diet and medication stabilization, baseline microbiome-based stratification, intervention periods of at least 12–24 weeks for immune biomarker readouts, longer follow-up for vaccine responsiveness or infection outcomes, and predefined responder/non-responder analyses. Primary endpoints should be clinically interpretable immune-aging markers rather than microbial abundance alone; secondary endpoints should include SCFAs, SBAs, tryptophan metabolites, intestinal permeability markers, inflammatory cytokines, and safety. These features should be in place before claims of a validated intervention for immunosenescence are made.

### 9.3. Synergistic Integration with Existing Anti-Aging Strategies

As microbiota-directed macromolecular modulators, MPPs may complement existing healthy-aging strategies by targeting immune metabolism, barrier integrity, inflammatory tone, and senescent-cell-associated pathways. A recent preprint reported that fucoidans can display senomorphic activity partly through sirtuin 6 (SIRT6)-dependent DNA-repair pathways [253]. Because fucoidans are marine algal polysaccharides, not MPPs, and the cited report has not yet undergone final peer-reviewed publication, this finding is included only as adjacent, hypothesis-generating evidence. LBP has also been reviewed in relation to oxidative stress, mitochondrial dysfunction, DNA damage, and cellular senescence [254], but these broad associations require validation with structurally defined fractions and aging-relevant causal models before combination-therapy claims are justified.

MPPs may also be investigated alongside strategies intended to improve immune resilience and vaccine responsiveness in older adults. By reshaping the aged gut microbiota and increasing immunoregulatory metabolites, defined preparations could plausibly support mucosal barrier function and reduce inflammaging; however, direct evidence that an MPP improves vaccine outcomes in older adults is not currently available. In a mouse study, *Aronia melanocarpa* polysaccharide altered adenosine monophosphate-activated protein kinase/sirtuin 1/nuclear factor κB and nuclear factor erythroid 2-related factor 2/heme oxygenase-1 signaling, together with inflammatory and microbiota-related readouts [255]. This adjacent preclinical evidence is hypothesis-generating and should not be extrapolated to standardized MPP products or human vaccination without direct testing.

**Figure 5:**
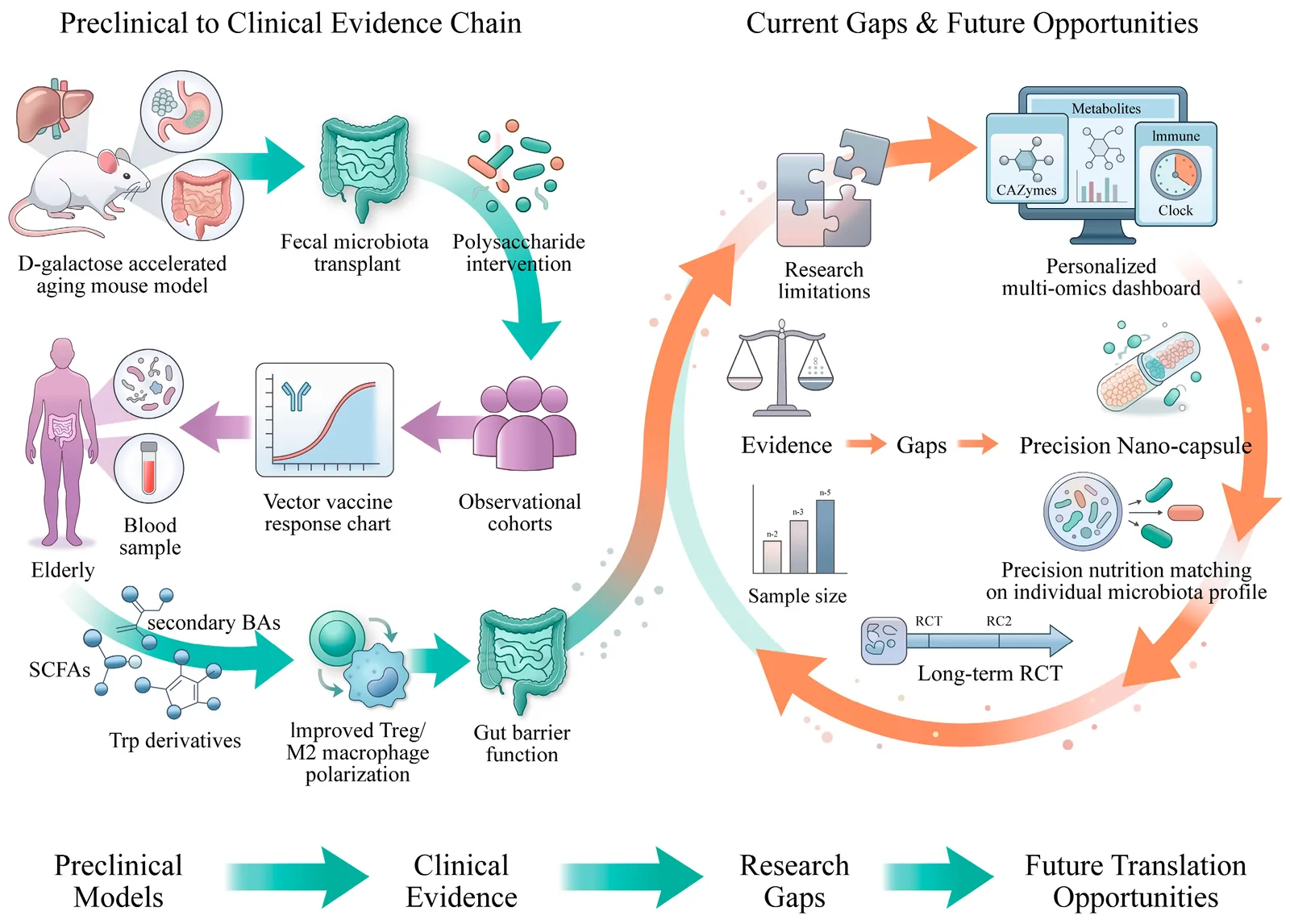
Translational roadmap for MPP-based immunosenescence interventions. The roadmap links preclinical mechanistic validation with clinical translation. Preclinical studies should define the causal sequence among MPP structure, microbial utilization, metabolite reprogramming, and immune-aging endpoints using aged animal models, microbiota depletion, FMT, gnotobiotic systems, metabolomics, and immune cell phenotyping. Human observational cohorts and RCTs should then evaluate whether MPP-driven changes in SCFAs, SBAs, and tryptophan-derived metabolites are associated with improved barrier function, reduced inflammaging, restored Th17/Treg balance, enhanced macrophage homeostasis, and improved vaccine or infection-related outcomes. Key current gaps include small sample sizes, short intervention durations, insufficient structural standardization, limited causal validation, and inadequate responder stratification. Future opportunities include microbiome-informed precision nutrition, multi-omics efficacy dashboards, colon-targeted delivery systems, personalized synbiotic formulations, and long-term RCT validation. Abbreviations: FMT, fecal microbiota transplantation; MPPs, medicinal plant polysaccharides; RCTs, randomized controlled trials; SBAs, secondary bile acids; SCFAs, short-chain fatty acids; Th17, T helper 17 cell; Treg, regulatory T cell. This schematic was created by the authors using Adobe Illustrator 2026 (Adobe Inc., San Jose, CA, USA); no panel, icon arrangement, or graphical composition was reproduced or adapted from a published source.

Evidence for combination use in sarcopenia, chronic inflammation, or autoimmune imbalance remains preliminary. In non-aging-specific cellular models, APS affected autophagy-related adenosine monophosphate-activated protein kinase/mechanistic target of rapamycin signaling in hepatocyte senescence, whereas a Lentinus edodes mycelial polysaccharide attenuated advanced glycation end-product-induced endothelial pyroptosis through a metastasis-associated lung adenocarcinoma transcript 1/microRNA-199b/mechanistic target of rapamycin–NLRP3-related axis (Figure 5) [256,257]. These examples support a broader translational model in which MPPs are not positioned as stand-alone anti-aging agents but as structurally tunable, microbiota-compatible components of multidimensional healthy-aging interventions. These in vitro findings may help prioritize mechanisms for future testing, but they do not demonstrate systemic anti-aging efficacy, clinical synergy, or benefit in older adults.

An updated search through 10 June 2026 identified additional evidence that further sharpens two boundaries of the field. First, recent experimental and synthesis work indicates that age-related intestinal T-cell dysfunction, barrier failure, microbiota remodeling, and impaired inflammation resolution form a bidirectional network rather than a unidirectional microbiota-to-immune pathway [258,259,260,261]. Second, recent polysaccharide literature converges on structure–target–microbiome or structure-selective frameworks and emphasizes batch definition, context-dependent fermentability, host-receptor engagement, and causal validation rather than class-wide efficacy claims [262,263,264,265]. These publications strengthen mechanistic plausibility but do not provide direct older-adult clinical evidence for any structurally defined MPP preparation; accordingly, the translational conclusions of this review remain deliberately cautious.

## 10. Conclusions

This review proposes and applies a structure-centered framework for understanding MPPs as microbiota-directed macromolecular modulators of immunosenescence. Unlike conventional views that emphasize direct immune stimulation, this framework highlights the capacity of structurally diverse MPPs, including β-glucans, arabinogalactans, pectic polysaccharides, and other plant-derived macromolecular carbohydrates, to serve as selective substrates for gut microbial degradation. Through CAZyme-dependent utilization and ecological cross-feeding, MPPs may reshape microbial communities and reprogram immunoregulatory metabolites, including SCFAs, SBAs, and tryptophan-derived indoles. These metabolites may influence immune-aging nodes through GPR43/GPR109A, AhR, FXR/TGR5, NLRP3, adenosine monophosphate-activated protein kinase/mechanistic target of rapamycin (AMPK/mTOR), and NF-κB signaling, thereby contributing to barrier support, Th17/Treg balance, macrophage polarization, inflammatory control, and systemic immune homeostasis. Most links in this chain remain mechanistic or preclinical rather than clinically validated in older adults.

Evidence from representative MPPs, including APS, GLP, and LBP, supports microbiota-, metabolite-, and immune-related effects primarily in preclinical models. Human evidence currently derives mainly from related non-digestible polysaccharides, conventional prebiotics, or polysaccharide-containing complex preparations and therefore provides only indirect translational support. However, the field remains constrained by incomplete causal validation, insufficient structural standardization, batch-to-batch variability, limited long-term clinical evidence, and substantial interindividual microbiome heterogeneity. Therefore, MPPs should currently be regarded as promising but not yet clinically established interventions for immunosenescence. Future progress will require rigorous structure–activity characterization, strain- and metabolite-level mechanistic validation, standardized quality control, microbiome-informed responder stratification, and large-scale RCTs with integrated multi-omics endpoints. With these advances, MPPs may become safe, accessible, and precision-compatible components of multidimensional strategies for promoting immune resilience and healthy aging.

## Figures and Tables

**Table 2:** Short-chain fatty acid production, signaling mechanisms, and relevance to immune aging.

Dimension	Key Points	Immune-Aging Relevance	Ref.
Fermentation profile	Acetate, propionate, butyrate, and valerate are the major SCFAs generated by microbial fermentation; their relative proportions depend on substrate structure and microbial composition. Major producers include *Bacteroides*, *Bifidobacterium*, *Akkermansia*, *Faecalibacterium*, *Roseburia*, Eubacterium, Megasphaera, and *Clostridium*.	Determines the magnitude and quality of downstream immunometabolic signaling.	[62,63]
GPCR signaling	SCFAs activate GPR41, GPR43, and GPR109A with ligand-specific potency and downstream signaling through cAMP, Ca^2+^, MAPK, and phospholipase C-related pathways.	Links microbial fermentation to immune cell activation, epithelial responses, and inflammatory tone.	[64,65]
HDAC inhibition	Butyrate is a potent HDAC inhibitor; propionate and valerate show moderate inhibitory activity, whereas acetate has weaker epigenetic effects.	Regulates chromatin accessibility and transcription of inflammatory and tolerance-related genes.	[66,67,68]
Treg regulation	Butyrate and propionate promote Foxp3^+^ Treg differentiation through HDAC inhibition and GPCR signaling.	Supports mucosal tolerance and suppresses excessive inflammatory responses.	[69,70]
Dendritic cells	Butyrate and propionate reduce dendritic cell (DC) maturation and pro-inflammatory cytokine production while promoting tolerogenic phenotypes.	Limits aberrant T-cell priming and supports immune tolerance.	[70,71]
Macrophages	Butyrate, propionate, and acetate suppress pro-inflammatory cytokine production and can inhibit NF-κB and NLRP3 inflammasome activation.	Attenuates macrophage-driven inflammaging and tissue inflammation.	[72,73,74]
Neutrophils	SCFAs regulate neutrophil chemotaxis, reactive oxygen species production, and apoptosis in a context-dependent manner.	May contribute to the resolution of inflammation but can show dose- and context-specific effects.	[75,76,77]
Intestinal epithelium	Butyrate enhances tight junctions, mucus production, antimicrobial peptide expression, and colonocyte energy metabolism; acetate and propionate also support barrier integrity.	Reduces epithelial permeability, microbial translocation, and endotoxemia-associated inflammaging.	[78,79,80]
Aging-related changes	Aging is generally associated with reduced SCFA-producing capacity, although preserved SCFA production is linked to healthier aging trajectories in some elderly and centenarian populations.	Declining SCFA availability may contribute to barrier dysfunction, immune dysregulation, and chronic inflammation.	[81,82,83,84]
Human and interventional evidence	High-fiber diets, inulin-type prebiotics, resistant starch, and prebiotic fiber interventions can increase SCFAs and improve selected immune or inflammatory parameters.	Provides translational support, although evidence specific to structurally defined MPPs remains limited.	[82,85,86,87]

**Table 3:** Secondary bile acid receptors, signaling pathways, and relevance to immune aging.

Dimension	Key Points	Immune-Aging Relevance	Ref.
Principal BA receptors	FXR, TGR5, vitamin D receptor (VDR), sphingosine-1-phosphate receptor 2 (S1PR2), pregnane X receptor (PXR), and retinoic acid receptor-related orphan receptor γt (RORγt) mediate BA-dependent signaling.	These receptors link microbial BA metabolism to inflammation, barrier function, lymphocyte differentiation, and metabolic homeostasis.	[97,98,99,100,101,102]
BA ligands	Primary BAs, including cholic acid and chenodeoxycholic acid, and SBAs, including DCA, LCA, and ursodeoxycholic acid, differ in receptor affinity and immunometabolic effects.	Ligand composition determines whether BA signaling is protective, neutral, or pro-inflammatory.	[97,98,99,103,104]
FXR signaling	FXR regulates BA synthesis through small heterodimer partner and fibroblast growth factor 15/19 signaling and modulates BA transporters in the liver and intestine.	FXR activation can suppress NF-κB and NLRP3 signaling, reduce inflammatory cytokine production, promote tolerogenic DC phenotypes, and protect intestinal barrier integrity.	[105,106,107,108,109,110]
TGR5 signaling	TGR5 activates cAMP/protein kinase A/cAMP response element-binding protein signaling and may also interact with AKT and ERK pathways.	TGR5 activation suppresses macrophage and DC inflammatory responses, reduces inflammasome activation, and supports epithelial barrier function.	[111,112,113,114,115]
VDR signaling	LCA-derived metabolites can activate VDR-dependent detoxification and immune-regulatory pathways.	VDR signaling suppresses Th17 differentiation, promotes Treg development, supports antimicrobial defense, and strengthens epithelial barrier function.	[99,116,117,118,119]
RORγt and S1PR2 crosstalk	DCA, LCA, 3-oxo-LCA, and 3-oxo-DCA can modulate RORγt activity, whereas S1PR2 contributes to BA-related inflammatory and lymphocyte trafficking pathways.	These pathways connect BA metabolism to Th17/ILC3 biology and inflammatory cell migration.	[102,120,121]
Aging-related BA changes	Aging is associated with altered primary and SBA pools, reduced receptor feedback regulation, impaired FGF19 signaling, and disrupted microbial BA metabolism.	Dysregulated BA signaling may contribute to inflammaging, metabolic dysfunction, and impaired mucosal immune regulation.	[10,122,123,124,125,126]
Therapeutic relevance	FXR agonists, TGR5 agonists, VDR ligands, and RORγt inverse agonists are being investigated for metabolic, inflammatory, and immune-mediated diseases.	These targets provide mechanistic reference points for evaluating MPP-induced BA remodeling.	[102,127,128,129]

**Table 4:** Tryptophan metabolic pathways, microbial indole metabolites, AhR signaling, and relevance to immune aging.

Dimension	Key Points	Immune-Aging Relevance	Ref.
Major tryptophan pathways	Tryptophan is metabolized through the kynurenine pathway, serotonin pathway, and microbial indole pathway. The kynurenine pathway predominates in host tissues, whereas indole derivatives are generated mainly by gut microorganisms.	Aging and inflammation often favor kynurenine metabolism, whereas microbial indoles are generally associated with barrier protection and immune regulation.	[137,138,139]
Microbial indole metabolites	Indole, IAA, IPA, indole-3-carbinol, IAld, indole-3-acetate, and skatole differ in microbial origin, AhR activity, and intestinal concentration.	Protective indole metabolites may support mucosal defense, whereas their depletion can contribute to inflammaging.	[139,140,141,142,143,144]
Host kynurenine metabolites	Kynurenine, kynurenic acid, quinolinic acid, xanthurenic acid, and 3-hydroxykynurenine have distinct neuroimmune and redox effects.	Increased kynurenine pathway activity is linked to chronic inflammation, immune suppression, oxidative stress, and neuroinflammatory aging.	[145,146,147,148,149]
AhR signaling	IAA, IPA, IAld, indole, and kynurenine can activate AhR with different potency and context-dependent outcomes.	AhR signaling regulates mucosal immunity, epithelial integrity, detoxification, and inflammatory tone.	[141,145,150]
AhR target genes and mediators	AhR activation regulates CYP1A1, IL-22, IL-10, Foxp3, RORγt-related programs, and antimicrobial peptides such as RegIIIγ.	These targets connect microbial tryptophan metabolism to barrier defense, immune tolerance, and Th17/Treg balance.	[141]
Effects on innate lymphoid cells	IAA, IAld, indole-3-carbinol, and IPA can promote IL-22-associated mucosal defense through AhR-dependent pathways.	Supports epithelial repair, antimicrobial defense, and mucosal resilience during aging.	[139,141]
Effects on T cells	Kynurenine and microbial indoles modulate Treg, Th17, Th1, Th22, and CD8^+^ T-cell responses in a ligand- and context-dependent manner.	Influences adaptive immune balance, inflammatory polarization, and T-cell exhaustion.	[145,151,152,153,154]
Effects on DCs	Kynurenine promotes tolerogenic DC phenotypes, whereas IPA may modulate mucosal DC responses.	Regulates T-cell priming, immune tolerance, and mucosal inflammatory thresholds.	[145]
Effects on intestinal epithelium	IPA, IAA, and indole enhance barrier integrity, mucus production, antimicrobial defense, and antioxidant responses through AhR-, PXR-, and related pathways.	Reduces epithelial permeability and limits endotoxemia-associated inflammaging.	[140,150,155]
Aging-related changes	Aging is associated with increased kynurenine-to-tryptophan ratio, elevated indoleamine 2,3-dioxygenase activity, and reduced levels of selected microbial indole derivatives, including IPA.	This metabolic shift may weaken antioxidant capacity, barrier function, and immune tolerance.	[10,156,157]

**Table 5:** Core biomarker domains recommended for evaluating immunosenescence in MPP intervention studies.

Domain	Priority Markers	Sample and Method	Interpretation for Immune Aging	Use in MPP Trials
Adaptive immune aging	Naïve/memory *CD4+* and *CD8+* T-cell subsets; CD28 loss; CD57 and KLRG1; TCR/BCR diversity	Peripheral blood flow cytometry; TCR/BCR sequencing	Captures thymic output decline, clonal expansion, immune exhaustion, and reduced vaccine competence.	Primary or key secondary endpoints in older-adult trials.
Regulatory/inflammatory balance	*Foxp3+* Treg cells, Th17 cells, Th17/Treg ratio, IL-10, IL-17A	Flow cytometry; cytokine assays	Reflects mucosal and systemic immune tolerance versus inflammatory skewing.	Mechanistic endpoint linking SCFA/indole shifts to T-cell homeostasis.
Innate immune activation	Monocyte/macrophage phenotype, neutrophil function, dendritic-cell activation, NK-cell activity	Flow cytometry; ex vivo stimulation; phagocytosis/cytotoxicity assays	Assesses inflammatory priming and age-related innate immune dysfunction.	Important for distinguishing immune support from nonspecific stimulation.
Inflammaging and inflammasome activity	*IL-6*, *TNF-α*, *IL-1β*, IL-18, hs-*CRP*, NF-κB-related transcripts, NLRP3/caspase-1 markers	Serum/plasma immunoassays; PBMC transcript/protein assays	Measures chronic inflammatory tone and inflammasome activation relevant to immunosenescence.	Should be prespecified rather than inferred from microbiota changes alone.
Barrier integrity and endotoxemia	*LPS*/LBP, zonulin, I-FABP, fecal calprotectin; tight-junction proteins such as *ZO-1*/occludin when tissue is available	Blood/stool assays; permeability testing; tissue assays in preclinical studies	Tests whether MPPs interrupt the leaky gut-endotoxemia-inflammaging axis.	Bridge endpoint connecting microbiota metabolites to systemic immune effects.
Microbiota and metabolites	Strain-level primary degraders; CAZyme/PUL capacity; SCFAs, SBAs, indoles and kynurenine/tryptophan ratio	Shotgun metagenomics; targeted metabolomics; functional fermentation assays	Determines whether immune effects track with functional microbial metabolism rather than taxonomy alone.	Essential secondary endpoint and responder-stratification variable.
Clinical immune resilience	Vaccine antibody response, infection incidence/severity, frailty or physical function measures, safety/tolerability	RCT follow-up; vaccine challenge substudies; adverse-event monitoring	Provides clinically interpretable evidence beyond surrogate biomarkers.	Required before MPPs can be claimed as validated tools for immunosenescence.

**Table 6:** Structured summary of preclinical evidence linking MPPs, microbiota, metabolites, and immune-aging-related outcomes.

Evidence Tier and Design	Examples	Readouts Assessed	Interpretive Value
Most direct preclinical evidence: naturally aged or accelerated-aging animals	UP360, WMPs, LBP fractions, CDPS, DOPs, TFPS [208,209,210,211,212,213]	Dysbiosis correction, α-diversity/SCFA changes, altered *Lactobacillus*- or *Bacteroides*-related taxa, oxidative and inflammatory markers	Supports biological plausibility for immune-aging modulation, but causality remains incomplete.
Causal microbiota evidence: antibiotic depletion, immunosuppression, FMT, or rescue strategies	CDPS and selected MPP-like interventions [212]	Loss of benefit after microbiota ablation or immune suppression; immune and cognitive/inflammatory readouts	Stronger than association; should become a minimum standard for key preclinical claims.
Mechanistic but indirect evidence: young inflammatory, tumor, metabolic, or colitis models	APS, GLP, GPS, Poria and other MPPs [206,207]	Taxonomic remodeling; SCFA, bile-acid, or tryptophan-metabolite shifts; Treg/Th17 balance; macrophage polarization; dendritic-cell function	Useful for mechanism discovery but should not be presented as proof of immunosenescence reversal.
Substrate-utilization evidence: in vitro fermentation, defined cultures, enzyme/PUL analyses	LBP, *Cordyceps* polysaccharide, *Dendrobium* polysaccharides and comparators [52,53,54,55]	Primary degrader selection, CAZyme/PUL activation, SCFA or indole outputs; usually no full immune-aging phenotype	Clarifies structure–microbiota links but requires in vivo validation.
Translational bridge: older-adult observational and exploratory intervention evidence	NPS vaccine adjuvant, GOS, plant extract studies [214,215,216,217,218,219,220]	*Bifidobacterium*/*Prevotella* response patterns, metabolite signals, vaccine seroprotection, common-cold incidence, metabolic or functional outcomes	Supportive but not yet direct evidence for standardized MPP clinical efficacy.

## Data Availability

No new data were created or analyzed in this study. Data sharing is not applicable to this article.

## Supplementary Materials

The following supporting information can be downloaded at https://www.mdpi.com/article/10.3390/nu18162641/s1, Supplementary Method S1: Detailed search strategy and evidence-appraisal criteria retained outside the main text for transparency; Table S1: Expanded representative MPP evidence matrix moved from Table 1 in the main text; Table S2: Minimum structural characterization and quality-control checklist; Table S3: Evidence-tier definitions for interpreting mechanistic, preclinical, and clinical findings.

## Abbreviations

The following abbreviations are used in this manuscript:

ABCs	Age-associated B cells
AG	Arabinogalactan
AhR	Aryl hydrocarbon receptor
AMP	Antimicrobial peptide
AMPK	Adenosine monophosphate-activated protein kinase
APS	Astragalus polysaccharides
ATP	Adenosine triphosphate
BA	Bile acid
BCR	B-cell receptor
BSH	Bile salt hydrolase
CAR-T	Chimeric antigen receptor T cell
CAZymes	Carbohydrate-active enzymes
CDPS	*Cistanche deserticola* polysaccharides
DCA	Deoxycholic acid
DCs	Dendritic cells
DOPs	*Dendrobium officinale* polysaccharides
FMT	Fecal microbiota transplantation
Foxp3	Forkhead box P3
FTIR	Fourier-transform infrared spectroscopy
FXR	Farnesoid X receptor
GALT	Gut-associated lymphoid tissue
GC–MS	Gas chromatography–mass spectrometry
GHs	Glycoside hydrolases
GLP	*Ganoderma lucidum* polysaccharides
GMP	Good manufacturing practice
GOS	Galactooligosaccharides
GPCRs	G-protein-coupled receptors
GPR109A	G-protein-coupled receptor 109A
GPR41	G-protein-coupled receptor 41
GPR43	G-protein-coupled receptor 43
GPS	Ginseng polysaccharides
HDACs	Histone deacetylases
HPAEC–PAD	High-performance anion-exchange chromatography with pulsed amperometric detection
HPSEC/GPC	High-performance size-exclusion chromatography/gel permeation chromatography
IAA	Indole-3-acetic acid
IAld	Indole-3-aldehyde
IECs	Intestinal epithelial cells
IFN-γ	Interferon-γ
IL	Interleukin
ILA	Indole-3-lactic acid
IPA	Indole-3-propionic acid
KLRG1	Killer cell lectin-like receptor G1
LBP	Lycium barbarum polysaccharides
LCA	Lithocholic acid
LPS	Lipopolysaccharide
MAMPs	Microbe-associated molecular patterns
MASLD	Metabolic dysfunction-associated steatotic liver disease
MCP-1	Monocyte chemoattractant protein-1
MPPs	medicinal plant polysaccharides
mTOR	Mechanistic target of rapamycin
MUC2	Mucin 2
NF-κB	Nuclear factor κB
NLRP3	NOD-like receptor family pyrin domain-containing 3
NMR	Nuclear magnetic resonance
NPS	Non-digestible polysaccharide
Nrf2	Nuclear factor erythroid 2-related factor 2
PAMPs	Pathogen-associated molecular patterns
PD-1	Programmed cell death protein 1
PPARγ	Peroxisome proliferator-activated receptor γ
PRRs	Pattern-recognition receptors
PULs	Polysaccharide utilization loci
PXR	Pregnane X receptor
RCTs	Randomized controlled trials
RG-I	Rhamnogalacturonan-I
RORγt	Retinoic acid receptor-related orphan receptor γt
ROS	Reactive oxygen species
S1PR2	Sphingosine-1-phosphate receptor 2
SARS-CoV-2	Severe acute respiratory syndrome coronavirus 2
SASP	Senescence-associated secretory phenotype
SBA	Secondary bile acid
SCFAs	Short-chain fatty acids
SIRT6	Sirtuin 6
SOD	Superoxide dismutase
TCR	T-cell receptor
TFPS	Camellia flower polysaccharides
TGR5	Takeda G-protein-coupled receptor 5
Th17	T helper 17 cell
Tim-3	T-cell immunoglobulin and mucin-domain-containing protein 3
TLR4	Toll-like receptor 4
TNF-α	Tumor necrosis factor-α
Treg	Regulatory T cell
UDCA	Ursodeoxycholic acid
UP360	Standardized mushroom-based polysaccharide-rich composition
VDR	Vitamin D receptor
WMPs	White mushroom polysaccharides
ZO-1	Zonula occludens-1
