# Supplementary material for: Medicinal Plant Polysaccharides as Microbiota-Directed Modulators of Immunosenescence: Structural Determinants, Metabolite Reprogramming, and Host Immune Regulation
Source: Nutrients. 2026 Aug 12;18(16):2641. doi: 10.3390/nu18162641 (PMC13516415; doi:10.3390/nu18162641)
Supplement: Supplementary file 1 [file nutrients-18-02641-s001.zip › nutrients-4465334-supplementary.pdf]

Supplementary Materials

Supplementary Method S1. Detailed literature-search and evidence-appraisal approach

2.1. Literature Search Strategy

A structured narrative review was conducted to identify and synthesize evidence concerning the structural characteristics, gut microbial utilization, metabolite remodeling, and immunoregulatory effects of MPPs, with particular emphasis on immunosenescence and inflammaging. Relevant literature published between January 2000 to 10 June 2026 was searched in PubMed/MEDLINE, Scopus, and the Web of Science Core Collection, and the final database search was completed on 20 April 2026. Earlier landmark publications were additionally considered when they were essential for establishing the conceptual, structural, microbiological, or immunological foundations of the field. The search strategy combined controlled vocabulary terms, where available, with free-text keywords covering four principal domains: medicinal plant polysaccharides, gut microbiota and microbial carbohydrate utilization, microbiota-derived metabolites, and immunosenescence or host immune regulation. Representative terms included “medicinal plant polysaccharide”, “herbal polysaccharide”, “traditional Chinese medicine polysaccharide”, “plant-derived polysaccharide”, “gut microbiota”, “gut microbiome”, “intestinal microbiota”, “carbohydrate-active enzyme”, “CAZyme”, “polysaccharide utilization locus”, “polysaccharide utilization loci”, “microbial fermentation”, “cross-feeding”, “short-chain fatty acid”, “secondary bile acid”, “tryptophan metabolite”, “indole derivative”, “immunosenescence”, “immune aging”, “inflammaging”, “intestinal barrier”, “regulatory T cell”, “Th17”, “macrophage polarization”, and “inflammasome”. These terms were combined using the Boolean operators AND and OR. A representative search expression was (“medicinal plant polysaccharide” OR “herbal polysaccharide” OR “plant-derived polysaccharide”) AND (“gut microbiota” OR “gut microbiome” OR “intestinal microbiota”) AND (“immunosenescence” OR “immune aging” OR “inflammaging” OR “host immunity”). Additional targeted searches combined individual polysaccharide names or botanical sources, including Astragalus, Panax ginseng, Lycium barbarum, Ganoderma lucidum, Poria cocos, and Polygonatum, with terms related to microbial degradation, carbohydrate-active enzymes, polysaccharide utilization loci, microbial metabolites, intestinal barrier function, and immune-cell regulation. The reference lists of relevant reviews, systematic reviews, and key original studies were manually examined to identify additional eligible publications. Only English-language articles were considered. Because relatively few studies have simultaneously linked defined MPP structures, microbial utilization pathways, metabolite remodeling, and immunosenescence, mechanistically relevant studies of microbial glycan degradation,

microbiota-derived metabolites, intestinal immunity, and aging were also included when they directly informed the proposed structure–microbiota–metabolite–immunity framework.

2.2. Inclusion and Exclusion Criteria

Peer-reviewed original investigations, systematic reviews, meta-analyses, and authoritative mechanistic studies were considered eligible when they provided evidence relevant to the structural properties, microbial utilization, metabolic consequences, or immunoregulatory effects of MPPs. Eligible structural studies characterized one or more properties such as monosaccharide composition, glycosidic linkage patterns, molecular weight distribution, branching architecture, uronic acid content, chemical modification, or higher-order conformation. Microbiota-related studies were included when they examined microbial recognition, degradation, fermentation, ecological cross-feeding, carbohydrate-active enzymes, polysaccharide utilization loci, or changes in microbial community composition and function. Studies investigating short-chain fatty acids, secondary bile acids, tryptophan-derived indoles, or other microbiota-associated immunoregulatory metabolites were also considered, particularly when these metabolic changes were connected to intestinal barrier integrity, regulatory T-cell and Th17-cell balance, macrophage polarization, dendritic-cell function, inflammasome activation, immune-cell senescence, inflammatory cytokine production, or systemic inflammaging. Human studies involving older adults and investigations using naturally aged or experimentally aged animals were regarded as the most directly relevant evidence for immunosenescence. Studies conducted in young disease models were retained when they provided direct mechanistic information on microbiota-dependent metabolism, intestinal barrier regulation, or immune-cell responses, but their findings were interpreted separately from aging-specific evidence. In vitro fermentation studies, defined microbial culture experiments, and cell-based investigations were included when they clarified substrate utilization, metabolite formation, receptor signaling, or causal immune mechanisms. Studies were excluded when they examined polysaccharides unrelated to medicinal or biologically relevant plant sources without providing transferable mechanistic information, reported only general antioxidant or immunostimulatory effects without microbiota-, metabolite-, barrier-, or immune-aging-related outcomes, lacked sufficient information regarding the polysaccharide preparation or experimental design, represented conference abstracts, editorials, letters, commentaries, patents, or other non-peer-reviewed materials, or duplicated data reported in another included publication. Studies of conventional prebiotics and non-medicinal dietary polysaccharides were used only as mechanistic comparators and were clearly distinguished from direct evidence concerning MPPs.

2.3. Study Selection and Data Extraction

The retrieved records were initially screened on the basis of titles and abstracts, after which potentially relevant publications underwent full-text assessment. Final inclusion was determined by the direct relevance of each study to MPP structure, microbial utilization, metabolite production, intestinal barrier regulation, host immunity, or aging-related outcomes. For each included study, information was extracted on the botanical

source and plant part, extraction and purification procedures, polysaccharide fraction and reported purity, monosaccharide composition, molecular weight, glycosidic linkage pattern, degree of branching, uronic acid content, chemical modification, and higher-order conformation, where available. Experimental characteristics were also recorded, including study design, participant or animal age, health status, intervention dose and duration, microbiota profiling method, affected microbial taxa or functional pathways, metabolite measurements, intestinal barrier endpoints, immune-cell phenotypes, inflammatory mediators, and aging-related outcomes. Particular attention was paid to approaches capable of strengthening causal inference, such as antibiotic-mediated microbiota depletion, germ-free or gnotobiotic models, fecal microbiota transplantation, in vitro fermentation with defined microbial communities, targeted or untargeted metabolomics, receptor inhibition, genetic perturbation, and rescue experiments. Evidence from human studies, aged-animal models, young disease models, in vitro fermentation systems, microbial culture experiments, and isolated cell systems was considered separately whenever possible so that mechanistic observations were not inappropriately extrapolated across different biological and translational levels.

2.4. Evidence Synthesis

The included evidence was synthesized using a structured narrative approach centered on the proposed structure–microbiota–metabolite–immunity axis. The synthesis first examined how monosaccharide composition, glycosidic linkage patterns, molecular weight, branching architecture, uronic acid content, chemical modifications, and higher-order conformation influenced microbial substrate recognition and degradation. Evidence concerning carbohydrate-active enzymes, polysaccharide utilization loci, primary degraders, and microbial cross-feeding was then integrated to evaluate how structurally distinct MPPs shaped microbial ecological and functional responses. Subsequent analyses focused on changes in short-chain fatty acids, secondary bile acids, tryptophan-derived indoles, and other immunologically relevant metabolites, together with their potential effects on intestinal epithelial integrity, Treg/Th17 balance, macrophage polarization, dendritic-cell activity, inflammasome signaling, and inflammatory tone. Evidence concerning individual MPPs was compared according to the completeness of the link between structural characterization, microbial utilization, metabolite remodeling, and immune-aging-related outcomes. A quantitative meta-analysis was not undertaken because of substantial heterogeneity in botanical sources, extraction and purification procedures, polysaccharide composition, structural characterization methods, experimental models, intervention regimens, microbiome analytical platforms, metabolomic approaches, and immune outcome measures. The purpose of the synthesis was therefore to provide an integrative mechanistic and translational interpretation rather than a pooled estimate of intervention efficacy.

2.5. Evidence Appraisal and Methodological Considerations

The available evidence was appraised according to its directness, methodological rigor, degree of polysaccharide structural definition, mechanistic completeness, reproducibility, relevance to aging, and translational value. Human intervention studies and well-controlled investigations

conducted in aged animals were given greater interpretive weight than evidence derived exclusively from young disease models, in vitro fermentation systems, or isolated cell experiments. Studies demonstrating a sequential relationship among a chemically characterized polysaccharide, a defined microbial utilization process, a measurable metabolite alteration, and a specific immune outcome were considered more informative than studies reporting only changes in microbial taxonomic abundance. For the comparative evidence tables, the evidence was descriptively classified as strong, moderate, or weak. Strong evidence referred to a relatively complete chain connecting polysaccharide structural characterization, microbiota modulation, metabolite remodeling, and defined immune endpoints, preferably supported by intervention-based, depletion, transplantation, receptor-blocking, or other causal experiments. Moderate evidence referred to reproducible microbiota-related and immune effects for which structural characterization, metabolite-level analysis, aging relevance, or causal validation remained incomplete. Weak evidence referred to preliminary findings based mainly on microbial compositional changes, general immunomodulatory outcomes, limited experimental replication, or indirect extrapolation from non-aging models. These categories were developed for qualitative comparison within the present narrative review and do not constitute a formal Grading of Recommendations Assessment, Development and Evaluation assessment. Standardized risk-of-bias instruments were not applied because of the broad mechanistic scope and substantial methodological diversity of the included literature; consequently, the evidence classifications should be interpreted as structured narrative judgments rather than quantitative estimates of certainty.

Table S1. Expanded representative MPP evidence matrix

No.	Polysaccharide	Source	Structural characteristics	Experimental models	Gut microbiota changes	Metabolite changes	Immune endpoints	Evidence level	Main limitations
1	APS	A. mongholicus / A. membranaceus	Glc/Gal/Ara-rich heteropolysaccharides; β-1,3-linked backbone; MW 10–1000 kDa	Ulcerative colitis, experimental autoimmune encephalomyelitis, and tumor models	Increased Bacteroides, Blautia, and Faecalibacterium; decreased Proteobacteria	Increased butyrate and total SCFAs; regulated bile acid metabolism	Restored Th17/Treg balance; promoted M2 macrophage polarization; improved dendritic cell function	Strong mechanistic evidence	Evidence is mostly derived from young animal models; structural characterization remains incomplete
2	GPS	P. ginseng	Gal/Ara/Rha-rich pectin-type heteropolysaccharides; MW 10–500 kDa	Tumor, type 2 diabetes mellitus, and ulcerative colitis models	Increased Akkermansia, Bifidobacterium, and Lactobacillus; decreased	Increased SCFAs; increased tryptophan/kynurenine ratio; altered bile acid profiles	Activated macrophages through Dectin-1; enhanced PD-1/PD-L1-	Strong mechanistic evidence	Direct clinical evidence remains insufficient

				pathogenic bacteria		related antitumor responses; regulated Treg/Th17 balance		
3	LBP	L. barbarum	Gal/Ara/Rha-rich acidic heteropolysaccharides; MW 10–200 kDa	Immunosuppression, tumor, and naturally aged mouse models	Increased Lactobacillus and Bifidobacterium; decreased Enterobacteriaceae	Increased SCFAs and butyrate	Enhanced macrophage phagocytosis; restored T-cell proliferation; increased natural killer cell activity	Moderate mechanistic evidence	Metabolite-level and aging-specific evidence remain limited
4	GLP	G. lucidum	Glc-dominant β-1,3-glucans; MW 100–2000 kDa	Colitis, tumor, and metabolic disease models	Increased SCFA-producing bacteria and Bifidobacterium; decreased pathogenic bacteria	Increased acetate and butyrate; regulated bile acid metabolism	Activated macrophages through Dectin-1; enhanced antitumor immunity; promoted T-cell proliferation	Moderate mechanistic evidence	Studies in aged models remain insufficient
5	Poria polysaccharides	Poria cocos	Glc-rich β-1,3-glucans; MW 10–100 kDa	Immunosuppression and tumor models	Modulated gut microbial composition; increased beneficial bacteria	Increased SCFAs	Activated macrophages; enhanced immune responses	Moderate mechanistic evidence	Mechanistic and aging-specific validation remains limited
6	Tremella polysaccharides	Tremella fuciformis	Gal/Man-rich acidic heteropolysaccharides; MW 50–500 kDa	DSS-induced colitis model	Altered gut microbial structure	Not clearly defined	Increased Foxp3⁺ T cells; enhanced Treg function; alleviated colitis	Moderate mechanistic evidence	Metabolite evidence is lacking
7	Eucommia	Eucommia	Ara/Gal-rich	Drosophila	Modulated	Altered	Prolonged	Moderat	Evidence from

	polysaccharides	ulmoides bark	polysaccharides; MW 10–50 kDa	aging and obesity-associated cognitive impairment models	microbiota; increased beneficial bacteria	tryptophan metabolites; remodeled metabolic profiles	lifespan; improved cognition; alleviated neuroinflammation	e to weak	aged mammalian models is limited
8	Yam polysaccharides	Dioscorea opposita	Glc/Man/Gal-containing heteropolysaccharides	Immunosuppression and diabetes models	Increased Lactobacillus; decreased Enterococcus	Increased SCFAs	Activated macrophages; enhanced immune function	Moderate mechanistic evidence	Mechanistic studies are not systematic
9	Polygonatum polysaccharides	Polygonatum sibiricum	Pectin-type heteropolysaccharides	Aging and immunosuppression models	Modulated gut microbiota	Not clearly defined	Improved immune function; enhanced antioxidant capacity	Weak to moderate	Evidence remains fragmented
10	Gastrodia polysaccharides	Gastrodia elata	Heteropolysaccharides	Neuroinflammation and immunoregulation models	Modulated gut microbiota	Not clearly defined	Provided neuroprotection; regulated immune responses	Weak mechanistic evidence	Evidence directly related to immunosenescence is lacking
11	Poria peel polysaccharides	Poria cocos peel	Ara/Xyl/Gal-containing heteropolysaccharides	Immunoregulation models	Increased Bifidobacterium and Lactobacillus	Increased butyrate	Enhanced immune function	Moderate mechanistic evidence	The number of studies remains limited
12	Morinda polysaccharides	Morinda officinalis	Ara/Gal/Rha-containing heteropolysaccharides	Aging models	Modulated gut microbiota	Altered SCFA profiles	Exerted anti-aging effects; enhanced immune function	Moderate mechanistic evidence	In-depth mechanistic studies are lacking
13	Acanthopanax polysaccharides	Acanthopanax senticosus	Glc/Ara-containing polysaccharides	Immunosuppression and tumor models	Increased Lactobacillus	Increased SCFAs	Activated macrophages; enhanced antitumor immunity	Moderate mechanistic evidence	Aged-model evidence remains insufficient

14	Angelica polysaccharides	Angelica sinensis	Ara/Gal/Glc-containing heteropolysaccharides	Immunosuppression and anemia models	Modulated gut microbiota	Not clearly defined	Improved hematopoietic function; enhanced immune responses	Moderate mechanistic evidence	Mechanisms of action remain incompletely defined
15	Hedysarum polysaccharides	Hedysarum polybotrys	Structurally similar to APS	Immunoregulation models	Modulated gut microbiota	Altered SCFA profiles	Enhanced immune function	Moderate mechanistic evidence	The number of available studies is limited

Table S2. Minimum structural characterization and quality-control checklist for MPP studies

Domain	Minimum information	Recommended analytical approaches
Source authentication	Latin binomial, authority when needed, plant/fungal part, voucher specimen, origin, harvest/processing conditions	Botanical authentication records; voucher deposition
Extraction and purification	Solvent, temperature, time, deproteinization, decolorization, dialysis/ultrafiltration, chromatography, drying method	Detailed protocol sufficient for batch replication
Purity and contaminants	Total carbohydrate, residual protein, phenolics, nucleic acids, ash/moisture, endotoxin, pesticide/heavy-metal/microbial contamination where relevant	Colorimetric assays, elemental/ash/moisture analysis, LAL/endotoxin assay, pharmacopeial contaminant tests
Monosaccharide composition	Molar ratio and hydrolysis conditions	HPAEC-PAD, GC-MS, or LC-MS after validated derivatization/hydrolysis
Molecular-weight distribution	Mw, Mn, dispersity, fraction profile	HPSEC/GPC, preferably with MALS and refractive-index detection
Linkage and branching	Glycosidic linkages, branching points, degree of branching	Methylation-GC-MS; 1D/2D NMR including HSQC/HMBC/COSY/NOESY as needed
Acidic/charged substituents	Uronic acid, methyl esterification, acetylation, sulfation, phosphorylation	Uronic-acid assays, FTIR/NMR, ion chromatography, elemental sulfur/phosphorus analysis
Higher-order conformation	Triple helix, aggregation, rheology, morphology, solubility	Congo red assay, CD, AFM, SEM/TEM, DLS, rheology

Batch release and fingerprints	Batch-to-batch similarity and activity-linked release criteria	FTIR/NMR/chromatographic fingerprints; predefined acceptance ranges

Table S3. Evidence-tier definitions used in the narrative synthesis

Tier	Definition	Typical evidence	Interpretive use
Strong	Relatively complete chain from defined structure to microbial utilization, metabolite alteration, and immune endpoint, preferably with causal testing.	Aged or accelerated-aging models, microbiota depletion/FMT/gnotobiotic studies, receptor blockade, metabolite rescue, or well-controlled human intervention with immune endpoints.	Can support cautious mechanistic and translational claims.
Moderate	Reproducible microbiota and immune effects but incomplete structural definition, metabolite resolution, aging relevance, or causality.	Young disease models, repeated preclinical studies, or human pilot data without complete omics/immune validation.	Supports biological plausibility but not confirmed immunosenescence modification.
Weak	Preliminary associations, general immune stimulation, incomplete polysaccharide characterization, or indirect extrapolation.	In vitro fermentation only, cell assays alone, single small preclinical report, or taxonomic shifts without functional/metabolite data.	Hypothesis-generating only; should not be generalized to immune aging.
